# Transcriptomic Profiles of *Zymomonas mobilis* 8b to Furfural Acute and Long-Term Stress in Both Glucose and Xylose Conditions

**DOI:** 10.3389/fmicb.2020.00013

**Published:** 2020-01-23

**Authors:** Shihui Yang, Mary Ann Franden, Xia Wang, Yat-Chen Chou, Yun Hu, Steven D. Brown, Philip T. Pienkos, Min Zhang

**Affiliations:** ^1^State Key Laboratory of Biocatalysis and Enzyme Engineering, Environmental Microbial Technology Center of Hubei Province, School of Life Sciences, Hubei University, Wuhan, China; ^2^National Bioenergy and Biosciences Centers, National Renewable Energy Laboratory, Golden, CO, United States; ^3^Biosciences Division, Oak Ridge National Laboratory, Oak Ridge, TN, United States; ^4^BioEnergy Science Center, Oak Ridge National Laboratory, Oak Ridge, TN, United States

**Keywords:** *Zymomonas mobilis* 8b, furfural, xylose, transcriptomics, microarray, directional mRNA-Seq

## Abstract

*Zymomonas mobilis* 8b is an ethanologenic bacterium engineered to utilize both glucose and xylose. The impacts of lignocellulosic hydrolyzate inhibitors on the growth of *Zymomonas mobilis* 8b have been investigated. However, the molecular responses of these inhibitors have not been completely elucidated yet. In this study, molecular responses to furfural were investigated using transcriptomic approaches of both chip-based microarray and a directional mRNA-Seq. Furfural acute shock time-course experiment with 3 g/L furfural supplemented when cells reached exponential phase and stress response experiment in the presence of 2 g/L furfural from the beginning of fermentation were carried out to study the physiological and transcriptional profiles of short-term and long-term effects of furfural on 8b. Furfural negatively affected 8b growth in terms of final biomass and the fermentation time. Transcriptomic studies indicated that the response of 8b to furfural was dynamic and complex, and differences existed between short-term shock and long-term stress responses. However, the gene function categories were similar with most down-regulated genes related to translation and biosynthesis, while the furfural up-regulated genes were mostly related to general stress responses. Several gene candidates have been identified and genetic studies indicated that expression of ZMO0465 and cysteine synthase operon ZMO0003-0006 driven by its native promoter in a shuttle vector enhanced the furfural tolerance of 8b. In addition, the relationship between microarray and mRNA-Seq was compared with good correlations. The directional mRNA-Seq data not only provided the gene expression profiling, but also can be applied for transcriptional architecture improvement to identify and confirm operons, novel transcripts, hypothetical gene functions, transcriptional start sites, and promoters with different strength.

## Introduction

Lignocellulosic biomass is an abundant renewable and sustainable resource, which is considered as an excellent source of sugars for microbial conversion into liquid fuels and valuable biochemicals. However, due to its natural recalcitrance, pretreatments are necessary to make carbohydrates available for subsequent enzymatic hydrolysis and microbial fermentation. During the deconstruction processes, various inhibitory compounds with strong inhibition on hydrolytic enzymes and fermenting strains are generated due to the partial over-degradation of lignocellulose. These inhibitors include weak acids (e.g., acetic acid), furan aldehydes such as furfural, and lignin degradation products (e.g., vanillin) (Jönsson et al., 2013). Among these compounds, furfural, derived from dehydration of pentose during biomass deconstruction, is one of the most prevalent inhibitors due to its high concentration in the hydrolyzates and strong toxicity to microorganisms (Heer and Sauer, 2008; Wierckx et al., 2011; Yang et al., 2018a). Although physical, chemical and biological inhibitor removal methods may facilitate substrate utilization and bioethanol fermentation, the removal of inhibitors from hydrolyzates before fermentation is not economical due to the loss of fermentable sugars and the cost associated with additional processing steps (Parawira and Tekere, 2011; Jönsson et al., 2013). To make economical lignocellulosic biofuels, it is very important to develop robust strains with high titer, yield and productivity in the presence of furfural and other inhibitors, and numerous efforts have been devoted to meeting this goal (Mills et al., 2009).

*Zymomonas mobilis* is a model ethanologenic facultative anaerobic strain with many exceptional characteristics, such as unique anaerobic use of the Entner-Doudoroff (ED) pathway that results in low cell mass formation and high-specific rate of sugar uptake and ethanol production (Panesar et al., 2006; Rogers et al., 2007; Wang et al., 2018). Other than ethanol production from pure sugars and lignocellulosic materials, *Z. mobilis* has been engineered for other biochemicals such as 2,3-butanediol and sorbitol, which could be engineered as an ideal microbial platform for future biomass biorefinery (Xia et al., 2019; He et al., 2014; Yang et al., 2016). In addition, the availability of genome sequences for multiple strains (Seo et al., 2005; Yang et al., 2009a; Zhao et al., 2012), metabolic modeling results (Widiastuti et al., 2011; Pentjuss et al., 2013), exogenous and native CRISPR-cas genome editing tools and biological part characterization methods for strain engineering (Kerr et al., 2011; Jia et al., 2013; Zhang et al., 2013; Shen et al., 2019; Yang et al., 2019a, b; Zheng et al., 2019) also help expedite the research progress in *Z. mobilis*.

Native *Z. mobilis* can only ferment sucrose, glucose, and fructose, but not pentose sugars like xylose and arabinose. To make *Z. mobilis* utilize xylose, which is the second most abundant sugar within lignocellulosic hydrolyzates, an engineered *Z. mobilis* strain 8b was constructed expressing heterologous genes of *talB*, *tktA*, and *xylAB* from *E. coli* for xylose utilization (Zhang et al., 2007). Consistent with the wild-type strain, *Z. mobilis* 8b is sensitive to furfural, and this inhibitory effect can be subtly exacerbated when grown in xylose (Franden et al., 2013).

To address this limitation, many efforts have been performed by traditional genetic engineering or by adaptive evolution strategy to improve the furfural tolerance in *Z. mobilis* strains (Dong et al., 2013; Mohagheghi et al., 2015; Shui et al., 2015; Tan et al., 2015; Wang et al., 2017; Yang et al., 2018c). Despite of these advances, much more work is still needed to achieve high ethanol yields from lignocellulosic biomass for a commercial process in the future. The accumulation of *Z. mobilis* systems biology data could help reveal the relationships between genetic features and inhibitory stressors under different conditions (Yang et al., 2009b, 2010; He et al., 2012b, 2014; Yang et al., 2013, 2016, 2018b; Mohagheghi et al., 2014), which could provide important guidelines for strain improvement.

A transcriptomic study has been conducted for furfural response in *Z. mobilis* wild-type ZM4 at stationary phase, and the analysis revealed that furfural had effects on multiple aspects of cellular metabolism at the transcriptional level (He et al., 2012b). However, this study was performed in pure glucose condition since ZM4 can only utilize glucose but not xylose. Since most biomass hydrolyzate contains a large amount of both glucose and xylose, and xylose has been identified as a stressor for *Z. mobilis* (Mohagheghi et al., 2014), we examined the transcriptional profiles of *Z. mobilis* 8b with xylose-fermenting ability to both glucose and xylose in short-term and long-term furfural stress conditions in this study. To gain more comprehensive insights into mechanisms of furfural toxicity and tolerance in *Z. mobilis*, our current transcriptomic study was conducted focusing on the effect and interaction of furfural in short-term and long-term furfural stress conditions, two carbon sources of glucose and xylose, as well as two different growth stages. The knowledge gap and furfural-responsive genes identified in this study will help unravel the underlying mechanism of furfural tolerance and provide genetic targets for robust industrial strain development.

## Results

### Physiological Responses to Furfural

Previous fermentation experiments (Mohagheghi et al., 2014) indicated that it took about 4∼6 h and 12 h for 8b to reach log and stationary phases reaching an OD_600 nm_ values of 0.8∼1.2 and 9.0 respectively in RMG8 from an initial OD_600 nm_ value of 0.1. It will take longer time for 8b to reach similar log and stationary phase in RMX8, which were 24∼30 h and more than 48 h with a corresponding OD_600 nm_ values of 0.7∼1.0 and 3.7, respectively.

The effect of furfural on 8b growth in RMG2 and RMX2 has been reported in a small-scale Bioscreen C assay (Franden et al., 2009, 2013). In order to characterize the effect of furfural on growth and fermentation of *Z. mobilis* 8b and further understand the effect of the furfural at cellular level using transcriptomics approaches, we conducted fermentations with the supplementation of various concentrations of furfural using *Z. mobilis* 8b. Our results showed that the growth rates were dramatically affected by the addition of exogenous furfural when either glucose or xylose was used as the carbon source (Supplementary Figure S1).

The concentrations of furfural, sugars and ethanol were also monitored for fermentation using glucose as the carbon source, which were then analyzed by HPLC and plotted along with cell densities against furfural concentrations (Supplementary Figure S2). The result indicated that even after furfural was consumed in fermentors with the supplementation of the 1 or 2 g/L furfural, growth rates remained lower than those without exogenous furfural supplementation. In addition to the decrease of specific growth rates, ethanol production and cell mass were greatly reduced with the increasing concentrations of furfural (Supplementary Figure S2). Growth stalled for 8b with the supplementation of 5 g/L furfural, and was very low in the presence of 4 g/L furfural. The furfural concentration of 3 g/L, at which level it could inhibit about 75% of the 8b growth rate (Supplementary Figure S2), was then selected for shock response experiment with pure furfural added into three replicate fermentors of F1, 2, 3 containing RMG8 and three fermentors of F7, 8, 9 containing RMX8 when they reached log phase with an OD_600 nm_ value around 1.0 (Table 1). The concentration of 2 g/L inhibiting about half of the 8b growth rate (Supplementary Figure S2) was selected for stress response experiment, which was supplemented into three fermentors of F4, 5, 6 containing RMG8 and three fermentors of F10, 11, 12 containing RMX8 before inoculation (Table 1).

**TABLE 1:** Experimental design and sampling time for furfural stress and shock responses.

Media	Fermentor#	Furfural treatment	Time points for transcriptomics	Time points for OD and HPLC
RMG8	F1, 2, 3	3 g/L added when reaching log phase	0, 15, 60 min post furfural shock	Every 2 h
RMG8	F4, 5, 6	2 g/L added from the beginning	Log and stationary	Every 4 h
RMX8	F7, 8, 9	3 g/L added when reaching log phase	0, 15, 60 min post furfural shock	Every 6–8 h
RMX8	F10, 11, 12	2 g/L added from the beginning	Log and stationary	Every 8–12 h

**FIGURE 1:**
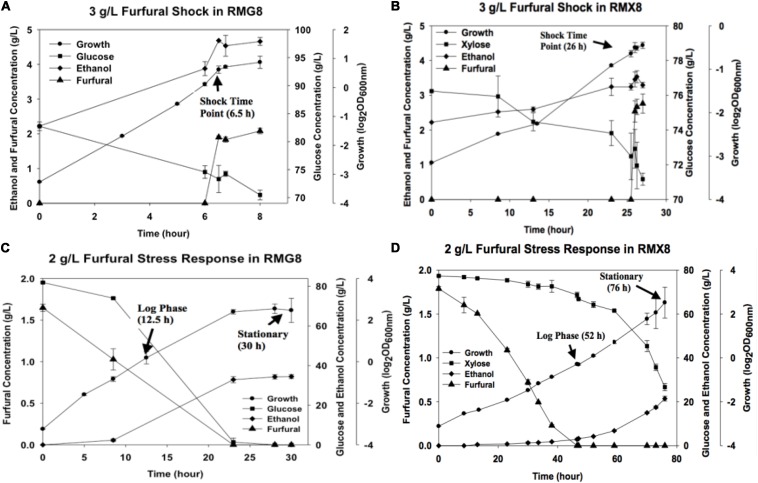
Furfural concentration, cell growth, sugar utilization, and ethanol production in RMG8 or RMX8 media with furfural shock or stress. **(A)** Time course shock response with the supplementation of 3 g/L furfural at log phase in RMG8; **(B)** time course shock response with the supplementation of 3 g/L furfural at log phase in RMX8; **(C)** stress response with the supplementation of 2 g/L furfural from the beginning of fermentation in RMG8; **(D)** stress response with the supplementation of 2 g/L furfural from the beginning of fermentation in RMX8.

The presence of furfural negatively affected growth in *Z. mobilis* 8b fermentations (Figure 1). Shock response experiment showed that the supplementation of furfural into fermentation media impeded the exponential growth of 8b in RMG8 and RMX8 with growth stalled immediately (Figures 1A,B). When furfural was added from the beginning of fermentation, the maximum biomass in terms of OD_600 nm_ values in different media (RMG8 and RMX8) were both about 6 but with longer time to reach the stationary phase, with an almost 12 h delayed in RMG8 and 30 h in RMX8 (Figures 1C,D).

Sugar consumption and the kinetics of ethanol and furfural concentrations were detected by HPLC and compared (Figure 1). Corresponding to the reduced growth, *Z. mobilis* 8b consumed sugar more slowly with furfural supplementation. As ethanol production was correlated with the consumption of glucose or xylose, ethanol accumulation became slower compared to control cells (Figure 1). In addition, for furfural conversion, almost no furfural was reduced in the shock response experiment for its short time duration within 60 min (Figures 1A,B). While in the furfural stress experiment, sugar appeared to be utilized mostly after most of furfural was converted, especially for xylose as the sole carbon source (RMX8), where furfural was consumed completely before cells reached the stationary phase (Figures 1C,D).

### Transcriptomic Profiling of *Z. mobilis* in Response to Furfural

Samples were taken at different time points post inoculation (Table 1 and Figure 1) and gene expression profiles were analyzed using a NimbleGen high density expression array as described previously (Yang et al., 2010). Two types of analysis were conducted: (1) time course shock response to investigate how furfural affects 8b in short term (from time zero without furfural in log phase (0 min) to 15 and then 60 min after furfural exposure with 3 g/L furfural added into fermentors); (2) stress response to study the long-term effect of furfural on 8b transcriptomic profiling including log and stationary phases with the presence of 2 g/L furfural from the beginning of the fermentation as well as log phase gene expression with or without the supplementation of furfural (Supplementary Table S1).

Before the application of the statistical modeling (Analysis of variance, ANOVA), the microarray data were evaluated using several approaches and the quality was found to be high. The correlation coefficients for biological replicates of microarray experiments were high, with *r* > 0.97 for each comparison (Supplementary Figure S3A). In addition, the biological replicates of microarray results were also grouped together closely based on the hierarchical clustering and principal components analyses (Supplementary Figures S3B,C), and the data can be grouped or separated by time points or the furfural treatment.

### Furfural Shock Response Transcriptomic Analysis

#### Overview of Sugar, Time-Course and Furfural Effects

To investigate the short-term furfural effects on 8b, 3 g/L furfural was supplemented into three fermentors containing RMG8 or RMX8 media when 8b reached exponential phase with an OD_600 nm_ around 1.0∼1.5 (Figure 1 and Table 1). Eighteen arrays were performed from samples taken at time points 0 before furfural supplementation as well as 15, 60 min post furfural shock (Supplementary Table S1). The overview of significantly differentially expressed genes is shown in Supplementary Figure S2. The number of differentially expressed genes in response to furfural or sugars is listed in Table 2, and the detailed gene expression information is summarized in Supplementary Table S3. Consistent with our previous acetate stress response transcriptomic study (Mohagheghi et al., 2014), the variable of sugar (glucose versus xylose) caused a dramatic transcriptomic changes. When xylose was used as the sole carbon source, 102 genetic features (including genes and intergenic regions from both annotated chromosome and plasmids) were significantly up-regulated and 71 were significantly down-regulated at least twofolds compared to that using glucose as the sole carbon source (Supplementary Figure S4A and Supplementary Table S3-8). Apparently, xylose itself is a stressor for 8b with stress response genes up-regulated and the biosynthesis genes such as ribosome proteins down-regulated (Supplementary Table S3-8).

**TABLE 2:** Summary of significantly differentially expressed genes in response to furfural or carbon sources in short-term shock response experiment.

	cGene	cIGR	pGene	pIGR	Total	Note
**Furfural regulated features**						
Gluc_15/0min-Up	24	9	0	0	33	Furfural regulated genes in RMG8 15 min post furfural shock, Supplementary Table S3-4.
Gluc_15/0min-Down	1	0	0	0	1	
Gluc_60/0min-Up	26	7	2	0	35	Furfural regulated genes in RMG8 60 min post furfural shock, Supplementary Table S3-5.
Gluc_60/0min-Down	6	3	0	0	9	
Xyl_15/0min-Up	3	0	0	0	3	Furfural regulated genes in RMX8 15 min post furfural shock, Supplementary Table S3-6.
Xyl_15/0min-Down	0	0	0	0	0	
Xyl_60/0min-Up	15	5	0	0	20	Furfural regulated genes in RMX8 60 min post furfural shock, Supplementary Table S3-7.
Xyl_60/0min-Down	24	11	0	0	35	
**Carbon-source regulated features**						
Xyl/Gluc_0min_Up	99	53	1	0	153	Xylose regulated genes compared to glucose without furfural shock, Supplementary Table S3-9.
Xyl/Gluc_0min_Down	109	52	0	0	161	
Xyl/Gluc_15min_Up	57	42	1	0	100	Xylose regulated genes compared to glucose 15 min post furfural shock, Supplementary Table S3-10.
Xyl/Gluc_15min_Down	68	32	1	0	101	
Xyl/Gluc_60min_Up	61	47	2	0	110	Xylose regulated genes compared to glucose 60 min post furfural shock, Supplementary Table S3-11.
Xyl/Gluc_60min_Down	52	20	0	0	72	

*cGene, number of chromosomal genes; cIGR, number of chromosomal intergenic regions; pGene, number of plasmid genes; pIGR, number of plasmid intergenic regions. Total, total number of genetic features including cGene, cIGR, pGene, and pIGR for either up-regulated or down-regulated genetic features.*

However, when furfural was supplemented, fewer genetic features were significantly differentially expressed in RMX8 compared to RMG8. At time zero before furfural supplementation, the utilization of xylose caused a substantial metabolic burden on 8b, with 99 genes up-regulated and 109 down-regulated (Table 2 and Supplementary Table S3-9), and these genes have much in common with that differentially expressed genes within 15 min and 60 min after furfural shock in RMX8 compared to RMG8 (Table 2 and Supplementary Tables S3-10, S3-11). This is consistent with our previous transcriptomic study of acetate stress response with xylose as the sole carbon source (Mohagheghi et al., 2014), which demonstrated that multiple stressor, such as xylose, furfural or acetate, could elicit the similar general stress responses.

Contrast to xylose responses, only a few genetic features were significantly differentially expressed in response to furfural when the effects of furfural in RMX and RMG were combined. Within the 15 min after furfural shock, 7 genetic features including 6 genes and 1 intergenic region were up-regulated with at least twofold increase and no down-regulated ones with at least twofold changes identified (Supplementary Figure S4B and Supplementary Table S3-2). During the time from 15 to 60 min post furfural shock, more genetic features were down-regulated although there were still a few in total (Supplementary Figure S4C and Supplementary Table S3-12). During 60 min after 8b exposure to furfural shock, four genes were down-regulated with at least twofold changes and 12 genetic features were up-regulated with at least twofold increase (Supplementary Figure S4D and Supplementary Table S3-3). Time-course transcriptomic study indicated that the response of 8b to furfural shock was fast and dynamic.

#### Transcriptomic Study of Genes Responsive for Furfural Resistance

**FIGURE 2:**
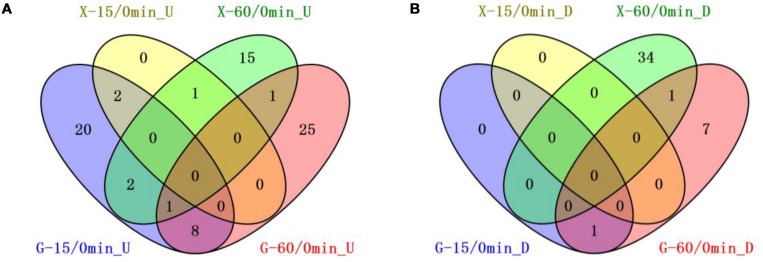
Venn diagrams of common genetic features shared by short-term furfural shock responses in RMG8 or RMX8. **(A)** The furfural up-regulated features after 15 or 60 min furfural shock with at least twofold increase; **(B)** the furfural down-regulated features after 15 or 60 min furfural shock with at least twofold decrease. **X-15/0min_U, G-15/0min_U**: genetic features up-regulated 15-min post furfural shock compared to 0 min in RMX8 or RMG8 medium respectively; **X-60/0min_U, G-60/0min_U**: genetic features up-regulated 60-min post furfural shock compared to 0 min in RMX8 or RMG8 medium respectively; **X-15/0min_D**, **X-60/0min_D**, **G-15/0min_D**, **G-60/0min_D** represent the down-regulated features in conditions same as above for up-regulated ones respectively.

We compared the 8b transcriptomic profiles in response to furfural in RMG8 or RMX8 to identify candidate genetic features related to furfural tolerance (Figure 2). Twenty-four chromosomal genes were up-regulated with at least twofold increase by furfural in RMG8 within 15 min after furfural shock (Table 2 and Supplementary Table S3-4). Four of these genes encode proteins of Fe–S assembly SUF system (ZMO0425-8) involved in sulfur metabolism. Only one gene (ZMO1437) encoding lysine transporter was down-regulated at this time with more than twofold changes. After 60 min post-shock, more genes were differentially expressed with 6 chromosomal genes down-regulated and 26 chromosomal genes up-regulated involving in amino acid metabolism and stress responses (Table 2 and Supplementary Table S3-5). In RMX8 medium, only three chromosomal genes were up-regulated with at least twofold increase and no genes down-regulated at 15 min post furfural shock (Table 2 and Supplementary Table S3-6). However, after 60 min post-shock, 15 chromosomal genes were found to be up-regulated by furfural, and these genes are mainly involved in sulfur metabolism and transport. Furfural also posed 24 chromosomal genes to be down-regulated in RMX8, which are mainly involved in transportation and transcriptional regulation (Table 2 and Supplementary Table S3-7).

The transcriptomic profiles of 8b at different times under same condition of carbon source (RMG8 or RMX8) were also investigated. In RMG8 medium, ten furfural responsive genetic features shared between the 8b cells with at least twofold changes within the total 34 genetic features differentially expressed by furfural at 15 min and 44 features at 60 min post furfural shock (Figure 2). However, in RMX8 medium, only one genetic feature was common between the ones after 15 and 60 min furfural shock with a total of 3 and 55 genetic features differentially expressed respectively (Figure 2). This indicated that the responses of 8b to furfural shock in RMG8 was faster than that in RMX8, since the differentially expressed genes already caused by the non-native xylose in RMX8 could help cells deal with the exogenous furfural supplementation.

Seven shared chromosomal genes up-regulated with at least twofold increase in RMG8 during the time-course after furfural shock are mainly involved in general stress responses and transportation (Figure 2A and Supplementary Tables S3-4, S3-5). ZMO1424 encodes ATP-dependent protease ClpB, and ZMO1588 encodes a UvrABC nucleotide repair system subunit UvrA. The up-regulation of these two genes in response to furfural could facilitate the repair of the damaged DNA and proteins to ensure normal cellular function. Other up-regulated genes encode the membrane efflux lipoprotein (ZMO0285), heavy metal transport protein (ZMO0916), TonB-dependent receptor (ZMO1298), ethanolamine transporter (ZMO1378), and a SnoaL-like protein (ZMO0174). ZMO1437 was the only down-regulated gene by furfural shared in RMG8 during the furfural shock, which is annotated as lysine exporter protein (Figure 2B and Supplementary Tables S3-4, S3-5). In RMX8 medium, ZMO1548 was the only shared up-regulated gene in response to furfural in 8b from 15 min to 60 min post-shock, which encodes squalene-hopene cyclase possibly involved in hopanoid biosynthesis (Figure 2 and Supplementary Tables S3-6, S3-7).

In addition, genes differentially expressed in RMG8 and RMX8 in response to furfural were compared as well to identify the common ones shared with different carbon sources (Figure 2). Within 15 min after furfural shock, ZMO0426 and ZMO0427 were the two up-regulated genes with at least twofold changes shared between those in RMG8 and RMX8 (Figure 2 and Supplementary Tables S3-4, S3-6). ZMO0426 and ZMO0427 encodes FeS assembly protein SufBD and SufS subfamily cysteine desulfurase respectively, which are involved in FeS assembly SUF system. Up-regulation of FeS assembly system may contribute to protecting 8b cells from oxidative stress caused by exogenous furfural supplementation (Lee et al., 2008; Jang and Imlay, 2010). Three ones shared by furfural in RMG8 and RMX8 at 60 min post-shock include two up-regulated genes ZMO0285 (encoding drug efflux lipoprotein) and ZMO0005 (encoding sulfate adenylyltransferase subunit, CysD), and one down-regulated gene ZMO1392 (encoding hypothetical protein) (Figure 2 and Supplementary Tables S3-5, S3-7). All genes shared at different times under different conditions discussed above are potentially responsive for the furfural tolerance in 8b cells and can be selected as genetic candidates for further investigation.

### Furfural Stress Response Transcriptomic Analysis

#### Overview of Sugar, Growth-Phase and Furfural Effects

Different from the furfural shock experiment, 2 g/L furfural was supplemented into three fermentors containing RMG8 or RMX8 media from the beginning with an initial OD_600 nm_ value around 0.1 to investigate the long-term furfural effects on 8b (Figure 1 and Table 1). Twelve arrays were performed from samples taken in log and stationary phases. In addition, six array data from exponential phase cells without furfural treatment were used as the control to compare the transcriptomic profiling of 8b with or without furfural treatment (Supplementary Table S1). Due to the complexity of the experiment involving variables of sugar, growth phase, and furfural treatment and our major interest on furfural responsive genes, we focused on the transcriptomic profiling in responding to furfural treatment. The number of differentially expressed genes in response to furfural is listed in Table 3, and the details of gene expression is included in Supplementary Table S4.

**TABLE 3:** Summary of significantly differentially expressed genes in response to furfural or carbon sources in long-term stress response experiment.

	cGene	cIGR	pGene	pIGR	Total	Note
**Furfural regulated genetic features**						
Gluc_Log_Fur/None-Up	30	16	0	1	47	Furfural regulated genes in RMG8 in the log phase, Supplementary Table S4.
Gluc_Log_Fur/None-Down	56	30	2	0	88	
Xyl_Log_Fur/None-Up	23	20	6	9	58	Furfural regulated genes in RMX8 in the log phase, Supplementary Table S4.
Xyl_Log_Fur/None-Down	100	43	1	0	144	
**Growth-phase regulated genetic features**						
Gluc_Fur_Stat/Log-Up	191	137	75	46	449	Furfural regulated genes in RMG8 from log phase to stationary phase, Supplementary Table S4.
Gluc_Fur_Stat/Log-Down	275	147	0	0	422	
Xyl_Fur_Stat/Log-Up	100	38	2	1	141	Furfural regulated genes in RMX8 from log phase to stationary phase, Supplementary Table S4.
Xyl_Fur_Stat/Log-Down	70	43	0	0	113	

*cGene, number of chromosomal genes; cIGR, number of chromosomal intergenic regions; pGene, number of plasmid genes; pIGR, number of plasmid intergenic regions. Total, total number of genetic features including cGene, cIGR, pGene, and pIGR for either up-regulated or down-regulated genetic features.*

In RMG8 medium with glucose as the carbon source, when 8b cells in the presence of furfural in log phase compared to that of without furfural, 47 genetic features (including genes and intergenic regions from both annotated chromosome and plasmids) were significantly up-regulated with at least twofold increase, and 88 were down-regulated with at least twofold changes (Table 3 and Supplementary Table S4). While in RMX8 medium, more genetic features were significantly differentially expressed in log phase comparison with or without the presence of furfural, 58 and 144 genetic features were significantly up-regulated and down-regulated with at least twofold changes respectively (Table 3 and Supplementary Table S4).

In addition, we also examined the effects of furfural on 8b gene expression profiles at different time phases, and found that much more genetic features were differentially expressed from log phase to stationary phase in the presence of furfural (Supplementary Table S4). Four hundred and forty-nine genetic features including 191 genes were up-regulated with at least twofold increase, and 422 genetic features including 275 genes were down-regulated with at least twofold decrease with furfural treatment in RMG8 between the log phase and stationary phases (Table 3 and Supplementary Table S4).

When the sole carbon source was xylose, furfural caused 141 genetic features up-regulated and 113 ones down-regulated with at least twofold changes when 8b entered the stationary phase from the log phase (Table 3 and Supplementary Table S4). However, most of these differentially expressed genes were not furfural specific and may be related to growth phase, since our previous studies discovered that dramatic differences existed at various stages of the growth phase with or without exogenous stressor supplementation (Yang et al., 2009b, 2013; Mohagheghi et al., 2014).

#### Transcriptomic Study of Genes Responsive for Furfural Resistance Shared Between Furfural Stress and Furfural Shock

Similar to furfural shock experiment, we firstly analyzed the transcriptomic profiles of 8b in the presence of furfural in RMG8 and RMX8 to identify candidate genes related to furfural tolerance. When the sole carbon source was glucose, 30 chromosomal genes were up-regulated by furfural in log phase compared to those without furfural supplementation (Supplementary Table S4). Ten of these are involved in sulfur metabolism including eight genes involved in cysteine synthesis (CysC, N, D, H, I, J, K < ZMO0003-5, 0007-9, 0748>, and ZMO0006), and two ABC transporter (SsuA, C < ZMO1261, 2>) are involved in sulfonate import. The 56 chromosomal genes down-regulated by furfural in RMG were involved in amino acid metabolism and transportation. When grown in RMX8 with xylose as the sole carbon source, furfural caused 23 chromosomal genes up-regulated involved in cell motility (for flagellar assembly) and chemotaxis, and 100 chromosomal genes up-regulated mainly involved in amino acid biosynthesis and metabolism (Supplementary Table S4).

**FIGURE 3:**
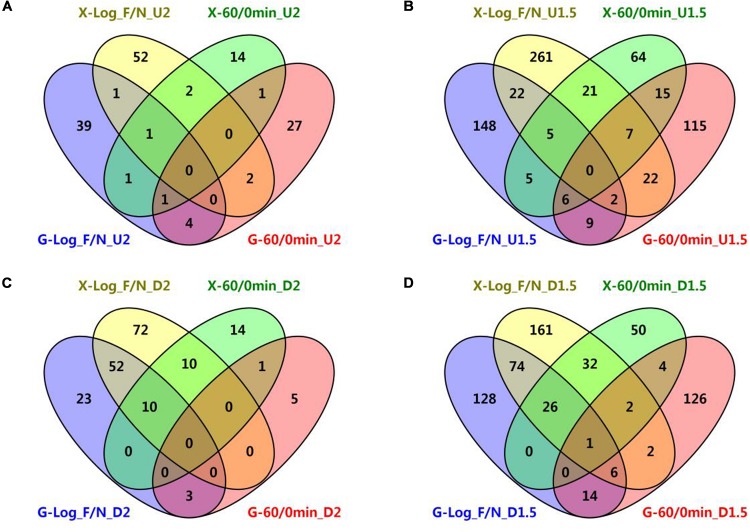
Venn diagrams of common genetic features shared by short-term furfural shock and long-term furfural stress responses in RMG8 and RMX8. **(A)** The furfural up-regulated features with at least twofold increase; **(B)** the furfural up-regulated features with at least 1.5-fold increase; **(C)** the furfural down-regulated features with at least twofold decrease; **(D)** the furfural down-regulated features with at least 1.5-fold decrease. **X-Log_F/N, G-Log_F/N:** genetic features up-regulated for long-term furfural stress in log phase compared to control without furfural supplementation in RMX8 or RMG8 medium respectively; **X-60/0min, G-60/0min:** genetic features up-regulated for short-term 60 min post furfural shock compared to control before furfural supplementation in RMX8 or RMG8 respectively; **U2, U1.5, D2, D1.5** represent significantly up-regulated 2 and 1.5-folds as well as down-regulated 2 and 1.5-folds respectively.

Genes shared under different conditions were also examined, and only two genes, ZMO0055 and ZMO0101, were up-regulated by furfural in both RMG8 and RMX8 (Figure 3A and Supplementary Table S4). ZMO0055 is annotated as hypothetical protein, and ZMO0101 as NAD-dependent epimerase/dehydratase. There were more common genes (39 chromosomal genes among total 62 shared genetic features) down-regulated by furfural between RMG8 and RMX8 (Figure 3C and Supplementary Table S4). However, most of these genes encode hypothetical proteins with two genes ZMO0560 (encoding aminotransferase) and ZMO0937 (encoding aromatic amino acid aminotransferase) involved in amino acid metabolism, and two other genes ZMO1522 and ZMO0128 are annotated as TonB-dependent receptors (Supplementary Figure S5).

In addition, the short-term and the long-term transcriptomic profiles of 8b in response to furfural were then taken into consideration to identify the common genes shared in both experiments. Since only three genetic features were found to be differentially expressed (up-regulated) in RMX8 15 min post furfural shock (Table 2 and Supplementary Table S3-6), our subsequent comparisons of differentially expressed genetic features were focused on results from 60 min post-shock and that from furfural stress experiment. However, different genetic features were involved in the short-term shock and long-term stress responses and only few genetic features had the similar differential expression (Figure 3). With glucose as the carbon source, five genes up-regulated with at least twofold increase shared between short-term and long-term furfural treatment, including ZMO1588 (encoding UvrA), ZMO2009 (encoding hypothetical protein), ZMO1457 (encoding MFS transport protein), and two cysteine synthesis related genes ZMO0004 (*cysN*) and ZMO0005 (*cysD*); three genes down-regulated with at least twofold decrease, including ZMO1437 (encoding lysine exporter protein) and two TonB-dependent transporter encoding genes ZMO0902 and ZMO0979 (Figure 3A and Supplementary Tables S3-5, S4).

While with xylose as the sugar, two genes encoding flagellar assembly (FliE, F < ZMO0632, 4>) and one encoding hypothetical protein (ZMO0055) were up-regulated by furfural in both short-term and long-term experiments, and 20 common down-regulated ones are mostly involved in transportation, such as ZMO1847 and ZMO1848 belonging to ABC transporter, ZMO1837 encoding molybdenum transport protein, ZMO1815 and ZMO1298 annotated as TonB-dependent transporter, and ZMO1378 annotated as ethanolamine transporter (Figure 3C and Supplementary Tables S3-7, S4). The transcriptomic analysis showed that these differentially expressed genetic features shared between the short-term and long-term experiments in RMG8 were different from those in RMX8 (Figures 3A,C). Even when the ratio of differentially expressed genetic features dropped to 1.5 folds with more genetic features included, there was only one common chromosomal gene ZMO0130 (encoding acid phosphatase) identified although the number of common ones shared respectively in RMG8 or in RMX8 increased as expected (Figures 3B,D). These results indicated that the effect of sugar played a crucial role on gene expression.

#### The Correlation Between Microarray and Directional mRNA-Seq

The breakthrough on next-generation sequencing technique development has opened the door for transcriptomic architecture investigation which cannot be achieved through traditional chip-based microarray technique. To explore the application of NGS-based mRNA-Seq on transcriptomics study, four samples from furfural shock time course experiment were selected including two samples before furfural shock at 0 min (control) and two samples at 15 min post-shock (treatment) from RMG fermentation experiment.

To fulfill the purpose of exploring mRNA-Seq technique and generating other transcriptomic information, the directional mRNA-Seq using Illumina Hiseq2000 was carried out with two control samples in one lane and two furfural-shocked samples in another lane. More than 40 million reads were generated for each sample with the length of 50-bp for each read, which has an average 1,000 times coverage of the whole 8b genome. The quality of mRNA-Seq results was checked using FastQC software and then imported into CLC Genomics Workbench 4.7 for RNA-Seq analysis to generate RPKM (reads mapping to the genome per kilobase of transcript per million reads sequenced) values for each genetic feature. The RPKM values of all genetic features of each individual sample were then imported into JMP Genomics 5.1 for statistical analysis. The quality control analysis indicated that there are good correlations among these biological replicates with *r* value greater than 0.97 (Supplementary Figure S6).

**FIGURE 4:**
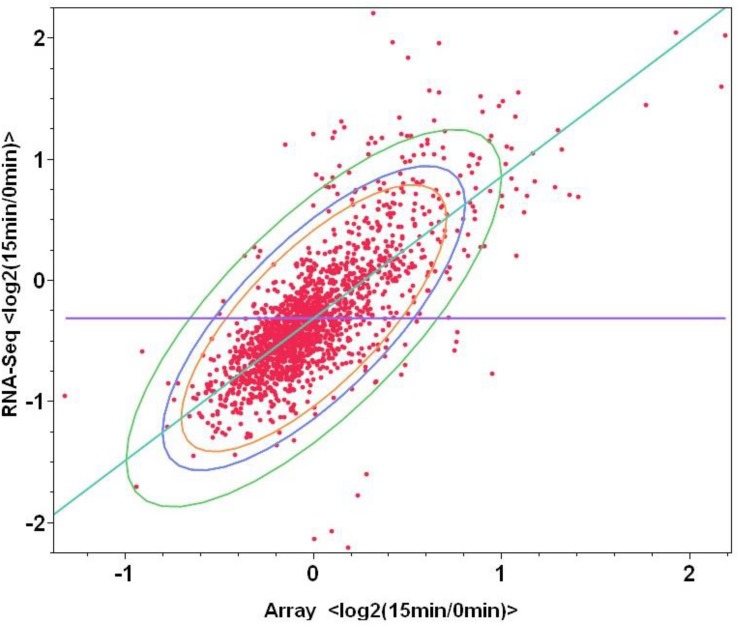
Correlations between two transcriptomic approaches of microarray and mRNA-Seq for the genetic feature changes in response to furfural shock. The *X*-axis and *Y*-axis are the log_2_-based gene expression differences after 15 min post furfural shock using microarray and RNA-Seq respectively.

To examine the correlations between microarray and mRNA-Seq to replace microarray in the future study which can provide more information than that of microarray, same total RNA samples were used for both microarray and mRNA-Seq experiments, log_2_-based gene expression value differences between the control (log phase samples before furfural shock: F1_0min and F2_0min) and treatment of 15 min post furfural shock (F1_15min and F2_15min) through microarray or mRNA-Seq were plotted against each other. When the values of all 1723 genetic features were used, the correlation between microarray and mRNA-Seq has a correlation number of 0.75, and the correlation increased to 0.84 when only the statistically significant data for 553 genetic features were used (Supplementary Table S5 and Figure 4), indicating the good correlation between these two transcriptomic platforms.

#### Improvement of Transcriptomic Architecture and Potential Application for Strain Improvement by RNA-Seq Data Mining

The directional mRNA-Seq can provide the reads number of each annotated genetic features representing the genetic expression level as what microarray does, since the data with good correlation and higher linear range with microarray data (Figure 4). More importantly, the directional mRNA-Seq can indicate the orientation of each transcript and therefore can be applied for annotation verification and identify novel ones without any previous annotation information (Supplementary Figures S7A,B). It can also help identify the transcription start site (TSS) (Supplementary Figure S7A), confirm the operon prediction (Supplementary Figure S7C), and potentially assign the function to novel transcript and hypothetical genes (Supplementary Figure S7D), which account for nearly one-third of all ORFs playing important roles in metabolism and stress responses and have been identified to be significantly differentially expressed in various experiment conditions for many microorganisms. Additionally, using the expression level of *xylAB* operon under strong promoter P*gap* as a reference, we can also identify the promoters with different strength (Supplementary Figures S7C,D), which can help future metabolic engineering practices to provide biological parts.

#### Characterization of Potential Furfural Responsive Genes Through Genetic Studies

Sulfur assimilation contributes to acetate tolerance and ethanol stress response in *Z. mobilis* (Yang et al., 2013; Mohagheghi et al., 2014). In the present study, several sulfur assimilation-related genes were up-regulated in the presence of furfural, especially in RMG8 medium. For example, all genes involved in cysteine synthesis (ZMO0003-9 and ZMO0748) and two ABC transporter involved in sulfonate import (ZMO1261-2) were up-regulated by furfural in RMG8 in the long-term stress response (Supplementary Table S4). Moreover, ZMO0004-5 and ZMO0008 were also observed up-regulated in response to furfural in the short-term shock experiment (Supplementary Table S3-1). Previous studies in *E. coli* and *S. cerevisiae* showed that many genes involved in sulfur assimilation into cysteine and methionine were up-regulated by furfural and exogenous addition of these two amino acids increased the inhibitor tolerance to furfural (Miller et al., 2009; Kanna and Matsumura, 2015). Thus, we investigated the effect of over-expression of an operon ZMO0003-0006 on furfural tolerance. In addition, ZMO0465 (*tpi*, triosephosphate isomerase) and ZMO0503 (glycosyl transferase family 2) were up-regulated with furfural stress when either glucose or xylose was used as the carbon source (Supplementary Table S4). These genes were then selected for genetics studies to investigate their roles on furfural tolerance.

Genes ZMO0465 and ZMO0503 as well as the operon ZMO0003-0006 were cloned into the pJL130-Sp plasmid, resulting in three constructs of pJL-13, pJL-14, and pJL-19 respectively, which were then electroporated into *Z. mobilis* after Sanger sequencing confirmation. Two recombinant strains were then obtained and further sequencing confirmed, which are 8b (pJL-13) and 8b (pJL-19) to over-express ZMO0465 and ZMO0003-0006 respectively. In addition, empty vector pJL130Sp was electroporated int 8b background and sequencing confirmed, which will be used as control strain for further genetics characterization. However, ZMO0503 can only be expressed within an *E. coli* background, but was apparently lethal when introduced into *Z. mobilis* since no transformants were obtained with a few times of electroporation experiments.

**FIGURE 5:**
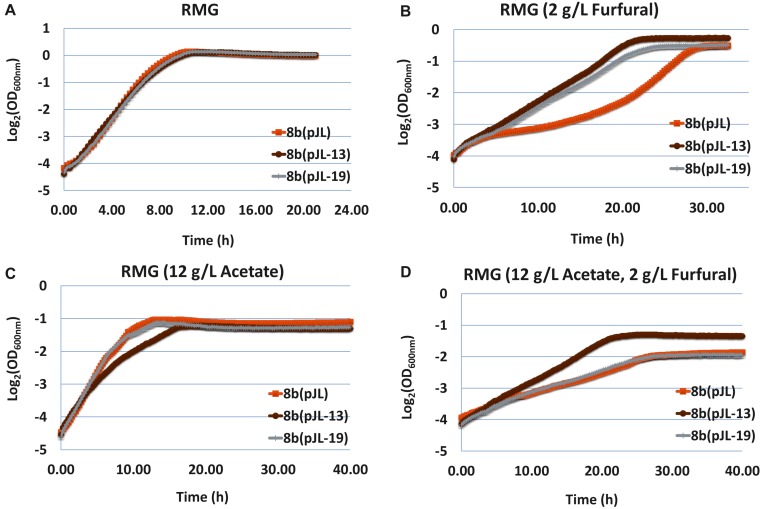
The growth of 8b (pJL) containing empty vector pJL130Sp as control and 8b derivative strains of 8b (pJL-13) and 8b (pJL-10) containing plasmid pJL-13 or pJL-19 to express operon ZMO0003-6 or ZMO0465 with its native promoter, respectively. The growth was monitored using Bioscreen C in RMG5 medium at 33°C **(A)**. Other conditions included RMG5 with the supplementation of 2 g/L furfural **(B)**, 12 g/L acetate **(C)**, as well as both 2 g/L furfural and 12 g/L acetate **(D)**. More than two independent experiments were performed with similar results, and triplicate was used in each experiment. Spectinomycin at a final concentration of 100 μg/mL was added into the media.

The furfural tolerance capability of these strains was evaluated in RMG medium containing classical hydrolyzate inhibitors of furfural and acetate as well as its combination. Compared with the control strain 8b (pJL) containing the empty vector pJL130Sp, 8b (pJL-13) and 8b (pJL-19) grew similarly with 8b (pJL) in RMG without any inhibitory compounds of furfural or acetate supplemented (Figure 5A). The growth of all strains became slow with the supplementation of either furfural (Figure 5B) or acetate (Figure 5C), but the impact of furfural and acetate was slightly different though. The lag phase of all strains increased and their final OD_600 nm_ values were higher with the addition of furfural (Figure 5B), while the final OD_600 nm_ values were greatly affected despite that the lag phase of these strains were only affected slightly with the supplementation of acetate (Figure 5C). The growth of all strains in medium with the supplementation of both acetate (12 g/L) and furfural (2 g/L) dramatically affected in terms of both extended lag phase time and decreased final OD_600 nm_ values (Figure 5D).

Recombinant strains containing plasmids to express ZMO0465 or ZMO0003-0006 grew better than the control strain containing empty vector pJL130Sp with the supplementation of furfural (Figure 5B). 8b (pJL-19) containing plasmid to express ZMO0003-0006 performed similarly to the control strain 8b (pJL) with the supplementation of acetate or the combination of acetate and furfural (Figures 5C,D). Interestingly, although the growth of 8b (pJL-13) containing plasmid to express ZMO0465 decreased in RMG with the supplementation of acetate, 8b (pJL-13) grew better than the control strain 8b (pJL) when both furfural and acetate were added into the medium. Since ZMO0465 cloned into pJL130SP for ZMO0465 expression involves in glycolysis for energy metabolism, the different growth patterns associated with 8b (pJL-13) and 8b (pJL-19) compared with the control strain 8b (pJL) indicated that these genes play different roles in cellular metabolisms and inhibitor tolerance, and the impact of different inhibitors in the cellulosic hydrolyzate was also different on microbial physiology (Figure 5).

## Discussion

Physiological experiments in both RMG8 and RMX8 showed that the presence of furfural severely inhibited the cell growth of 8b, leading to the decreased sugar consumption and ethanol production (Figure 1). In order to unravel the mechanism underlying these observations, the transcriptomic data of genes involved in metabolic pathways including xylose metabolism, glycolysis, and ethanol fermentation in *Z. mobilis* 8b were firstly investigated for xylose and furfural response in both furfural shock and furfural stress conditions (Supplementary Table S5).

Consistent with our previous acetate stress response transcriptomic study (Mohagheghi et al., 2014), xylose as the sole carbon source is a stressor for 8b which could cause the acetate accumulation leading to the buildup of NADH reducing equivalents and redox imbalance. Comparing cells grown in RMX8 versus RMG8 without furfural supplementation, aldehyde dehydrogenase SsdA encoded by ZMO1754, which catalyzes the conversion of acetaldehyde to acetate, was up-regulated 4 folds, and an iron-containing group III alcohol dehydrogenase (AdhA) encoded by ZMO1236 involving in oxidization of ethanol into acetaldehyde (Kalnenieks et al., 2002; Kalnenieks et al., 2006) was also up-regulated about threefolds (Supplementary Tables S5-1, S5-2). In addition, the excessive buildup of NADH in RMX8 could drive the reaction of xylose conversion toward the accumulation of xylitol, with two reductase genes ZMO0976 and ZMO1984 proposed to convert xylose to xylitol up-regulated with at least twofold changes (Agrawal and Chen, 2011).

However, when furfural was supplemented in RMX8 or RMG8, even the obvious inhibition has been observed in the physiological responses, almost all genes involved in sugar metabolism were shown to be subtly up-regulated as expected but at levels not considered significant, whether in the short-term shock response (Supplementary Table S5-1) or in the long-term stress response (Supplementary Table S5-2). A similar phenomenon has also been observed for *Z. mobilis* grown with exogenous furfural or ethanol supplementation (He et al., 2012a, b). Actually, previous study have identified that furfural could directly act on key enzymes of glycolysis and ethanol production, like pyruvate dehydrogenase (PDH) and alcohol dehydrogenase (ADH) by a non-competitive inhibition and competitive inhibition respectively (Modig et al., 2002). In addition, as shown in Figures 1C,D for furfural stress responses, the lower concentration of furfural in RMG8 (0.75 g/L) or RMX8 (0 g/L) in the log phase may also help elucidate its subtle negative effects on sugar metabolic pathway.

Studies for *E. coli* and *S. cerevisiae* showed that furfural can be converted into less toxic furfuryl alcohol by multiple NADH- and/or NADPH-dependent oxidoreductases, which actually is the primary step to eliminate its toxic effect on microbes (Liu et al., 2008; Miller et al., 2009). This principle is suitable for *Z. mobilis* strains (Franden et al., 2009), and some oxidoreductase genes were also identified in our present study to be differentially expressed by furfural which are supposed to be responsible for the inhibitor reduction conversion (Supplementary Table S5). For example, ZMO1696, encoding zinc-binding alcohol dehydrogenase which has been reported to be induced by phenolic aldehydes in *Z. mobili*s (Yi et al., 2015), kept up-regulated with more than 1.5-fold changes from 15 to 60 min after furfural shock in both RMG8 and RMX8 media (Supplementary Table S5-1). A similar up-regulation was detected for the aldo/keto reductase gene ZMO1984.

However, both the ZMO1696 and ZMO1984 were observed to be induced by xylose when 8b cells grown in RMX8 versus RMG8 without furfural supplementation. The result indicated that these furfural responsive oxidoreductase genes are non-specific, and 8b cells can regulate these non-specific genes promptly to degrade toxic furfural to alleviate its inhibitory effect. Although we have identified some genes supposed to be responsible for furfural degradation, some other oxidoreductase genes, such as ZMO0976 and ZMO1771, which have been confirmed to be able to catalyze furfural reduction by enzymatic assay, were not differentially expressed in this study (Agrawal and Chen, 2011; Wang et al., 2017). Thus, future works need to be performed by expression and knockout of these genes to further examine the relationship between the differentially expressed genes and furfural detoxification.

Our previous studies indicated that furfural resistance of *Z. mobilis* was increased in knockout mutants of either one of the ZMO0282, ZMO0283, and ZMO0285 genes encoding the RND efflux pump (Yang et al., 2014). ZMO0285 was reported to be downregulated 24 h post furfural stress (He et al., 2012b), and our study also indicated that ZMO0285 and ZMO0283 were down-regulated post furfural shock when either glucose or xylose was used as the carbon source (Supplementary Table S2). Since ZMO0964-0966 are paralogs of ZMO0282-0285, and ZMO0964 was similarly upregulated by furfural shock (Supplementary Table S2), it will be interesting to investigate the role of efflux pump on furfural tolerance. It was suggested that ZMO0281 and ZO0963 encoding TetR family transcriptional regulators controlling the efflux pump ZMO0281-0285 and ZMO0964-0966 respectively. In addition, the Blast result indicated that besides ZMO0281 and ZMO0963, another potential TetR family transcriptional regulator could be ZMO1547, although there is no adjacent efflux pump system associated with it. ZMO1547 can also be selected for further genetic studies.

In addition, other proteins involved in stress responses, including the unfolded protein response, oxidative stress, DNA damage and nutrient starvation, were also differentially expressed by furfural supplementation under different conditions. SUF system encoding genes ZMO0425-8 were up-regulated in RMG8 and RMX8 at 15 min after furfural shock, which are involved in FeS assembly and protect cells from oxidative stress caused by furfural (Lee et al., 2008; Jang and Imlay, 2010). ATP-dependent Clp protease encoding genes ZMO0405 (*clpA*) and ZMO1424 (*clpB*) kept up-regulated in RMG8 after furfural shock and contributed to degrading abnormal and damaged proteins resulted from furfural supplementation (Ambro et al., 2012). Nucleotide repair system UvrABC-related gene ZMO1588 (*uvrA*) was observed to be up-regulated in RMG8 both in furfural shock and furfural stress responses and its up-regulation will help repair the damaged DNA and proteins to ensure normal cellular function. Transcriptomic analyses confirmed that the general stress responses are crucial for furfural tolerance.

Our study also suggested that although transcriptomics analysis itself can be a powerful strategy to help identify candidate genes for genetics studies and to understand the global gene responses especially for microorganisms without established genetics system(s), classical genetics study is essential and should be included to verify the association of genotypic changes with phenotypic differences such as the relationship between the physiological responses of *Z. mobilis* to furfural and differentially expressed genes at different time points in this study. Although our study showed that the expression of an extra copy of ZMO0465 and cysteine synthase operon ZMO0003-0006 driven by its native promoter in a shuttle plasmid enhanced the furfural tolerance of 8b (Figure 5), which is consistent with previous study that up-regulation of genes involved in sulfur assimilation into cysteine and methionine increased the inhibitor tolerance to furfural (Miller et al., 2009; Kanna and Matsumura, 2015). Considering the fact that only a few gene targets that have been investigated, hypotheses related to other candidate genes are still needed to be evaluated, which is however time-consuming and probably impossible to achieve the goal in a short time using the classical genetics approaches since hundreds of gene targets and their combination need to be investigated. Therefore, advanced approaches such as CRISPRi/a for massive genetics study are needed to help elucidate the association of genotypic changes with phenotypic differences efficiently and effectively.

In summary, the physiological responses of a xylose utilization recombinant strain *Z. mobilis* 8b to short-term furfural shock and long-term furfural stress with both glucose and xylose as substrate were investigated in this study, and the presence of furfural reduced the cell growth, substrate utilization and ethanol production of 8b. The transcriptomic profiles of furfural shock and stress responses indicated that the molecular response of 8b to furfural was dynamic and complicate, and different genetic features were involved in the short-term shock and long-term stress responses. Several gene candidates have been identified and genetic studies indicated that expression of ZMO0465 and cysteine synthase operon ZMO0003-0006 driven by its native promoter in a shuttle plasmid enhanced the furfural tolerance of 8b, although further studies are still needed to explore the furfural tolerance mechanism and improve the strain inhibitor tolerance capability. In addition, different transcriptomics platforms of chip-based microarray and NGS-based directional mRNA-Seq have been explored and compared with good correlations with mRNA-Seq providing more information on transcriptional architecture.

## Materials and Methods

### Bacterial Strain and Growth Conditions

*Zymomonas mobilis* 8b was revived from frozen glycerol stocks in 10 mL RMG2 (2% glucose, 10 g/L yeast extract, 2 g/L KH_2_PO_4_) at 33°C for about 6∼8 h prior to inoculating overnight seed cultures at 33°C at 120 rpm in RMG5 (5% glucose) using shake flasks filled to 80% capacity. When the glucose concentration reached 20∼40 g/L, cells were spun down at 3,840 × *g* for 10 min at room temperature (RT) and resuspended in either RMG8 (8% glucose) or RMX8 (8% xylose) at a 10-fold concentration and used as inocula for fermentation studies.

### Plasmid Constructs for Candidate Gene Expression

The shuttle vector pJL130-Spec (Supplementary Figure S8) was used to investigate the effect of expressing candidate gene with its native promoter, which has been deposited into Genbank with the accession number of MN723559. The *Ptac* promoter and gene encoding Evoglow protein was replaced by candidate gene or operon with its native promoter. The primer sets used to PCR candidate gene/operon with its native promoters are: ZM0003_CF (CGAGCGGCCGCTGCAGAGATCTGAGCACGACCTCCCTT ATCA) and ZM0003_CR (AGCGGCCGCGAATTCGGTACCAA TGACTGGATCCGCTTGTC) for the operon of ZMO0003-6 and its native promoter with an expected 4966-bp PCR product; ZM0465_CF (CGAGCGGCCGCTGCAG AGATCTAAGCCGTTTTTGGTCATACG) and ZM0465_CR (AGCGGCCGCGAATTCGGTACCTCGAGAGGCGAGGTAAA CAG) for gene ZMO0465 and its native promoter with an expected 1565-bp PCR product; ZM0503_CF (CGAGCGGCCGCTGCAGAGATCTAAATGAAGATCGGGAT GCAG) and ZM0503_CR (AGCGGCCGCGAATTCGGTACCC GATAATCATGGCTTCAGCA) for gene ZMO0503 and its native promoter with an expected 1130-bp PCR product. The underline nucleotides containing restriction enzyme sites of *Not*I-*Pst*I-*Bgl*II for CF primers, and *Not*I-*Eco*RI-*Kpn*I for CR primers. Two primer sets were also designed for colony PCR and Sanger sequencing verification, which are 130_SF (GGGGCGCGCCATAACTTCGT) and 130_SR (AGCACAAGTTTTATCCGGCC), 130_SF2 (TCACCAGCTCACCGTCTTTC) and 130_SR2 (CCTGATG AATGCTCATCCGG). The schemes of the genetic fragments as well as details of the promoter regions of above three constructs were illustrated in Supplementary Figure S9. Promoter was predicted using the online server BPROM^1^. Genbank accession numbers for the *Z. mobilis* 8b genome used in this study are: CP023682.1 for chromosome, and CP023683.1, CP023684.1, CP023685.1, CP023686.1 for four native plasmids respectively.

NEB C2925 competent cells (NEB, Ipswich, MA, United States) were used for *E. coli* transformation, and transformants were confirmed by colony PCR using the primers of 130_SF/SR. The gene-specific primers were used to confirm that the targeted genes were cloned into the vector. Colonies showing the expected PCR bands were inoculated into LB broth supplemented with 50 μg/mL spectinomycin overnight and the plasmids were extracted and confirmed by PCR, restriction digestion, and Sanger sequencing.

### Electroporation Transformation

*Zymomonas mobilis* 8b cells were transformed with different plasmids by electroporation (Bio-Rad Gene Pulser, 0.1-cm gap cuvettes, 1.6 kV, 200 ohms, 25 μF). After electroporation, 1-mL RM medium was added to the electroporation mixture and cells were revived at 30°C for 3∼5 h. The revived culture was plated on RMG2 agar plates containing 100 μg/mL spectinomycin, and then incubated at 30°C for 2∼3 days. Transformants with expected PCR product size that were screened by colony PCR were then further confirmed by Sanger sequencing.

Electrocompetent *Z. mobilis* 8b cells were prepared by centrifuging cells from cultures that had reached an OD_600 nm_ value about 0.4∼0.6. Cell pellets were washed once in ice-cold sterile water, re-centrifuged, and washed twice in 10% glycerol. These pellets were resuspended in 10% glycerol at a concentration approximately 1,000-fold higher than that of the starting culture. Competent cells were stored at −80°C as small aliquots for later use.

### Bioscreen C Growth Assays

*Zymomonas mobilis* was revived from frozen glycerol stocks for 6∼8 h in 10 mL of RMG2 (2% glucose) at 33°C. Bioscreen C assays were carried out as described previously (Franden et al., 2009). Cells were inoculated into Bioscreen C wells containing a total volume of 300 μL and incubated without shaking at 33°C at an initial OD_600 nm_ value of 0.05 (approximately 5 × 10^6^ cells/mL). Turbidity measurements (OD_420__–__580 nm_) were taken every 10 min for up to 48 h.

### Controlled Batch Fermentations

Fermentations were performed in BioStat-Q Plus fermentors (Sartorius Stedim Biotech, France) using *Z. mobilis* 8b at 300-mL working volumes. RMG8 or RMX8 media were used for fermentation. The fermentors were inoculated at an initial OD_600 nm_ unit of 0.1. The fermentation was conducted at 30°C, 150 rpm, pH 6.0 with 2 N KOH for pH control. Fermentors were sparged with filter-sterilized nitrogen gas prior to fermentation. Samples were harvested during fermentation at different time points for transcriptomics study and HPLC analyses as described previously (Yang et al., 2009b). The details of experimental design including media, furfural concentration and sampling time points were listed in Table 1.

### High Performance Liquid Chromatography (HPLC)

Concentration of furfural, glucose, xylose, acetate, and ethanol were determined by HPLC from 0.2 μm-filtered samples taken at different time points during fermentation using Agilent1100 series HPLC (Agilent, CA). BioRad HPX-87H organic acids column and Cation H^+^ guard cartridge (Bio-Rad, CA) were used in this study with an operation temperature of 55°C. A refractive index detector (RID) was used for compound detection. Dilute sulfuric acid (0.01 N) was used as the isocratic mobile phase at a flow rate of 0.6 mL/min, following published procedures (Chen et al., 2016).

### RNA Extraction and ds-cDNA Synthesis

Total RNA was isolated from the cell pellet resuspended in TRIzol reagent (Invitrogen, CA) as described previously (Yang et al., 2009b). Briefly, RNase-free DNase I (Ambion, Austin, TX, United States) was used for each total RNA sample to digest residual genomic DNA, total RNA was subsequently purified using the RNeasy Mini Kit (Qiagen, CA). NanoDrop ND-1000 spectrophotometer (NanoDrop Technologies, Wilmington, DE, United States) was used to quantify the total cellular RNA, and Agilent Bioanalyzer (Agilent, CA) was used to assess RNA quality. Purified RNA with high quality was then used as the template to generate ds-cDNA using Invitrogen ds-cDNA synthesis kit (Invitrogen, CA).

### Microarray Sample Labeling, Hybridization, Scan, and Statistical Analysis of Array Data

Ds-cDNA labeling, hybridization, wash, and scan followed the NimbleGen protocols. A 12-bay hybridization station (BioMicro Systems Inc., Salt Lake City, UT, United States) was used for hybridizations, a Maui wash system (BioMicro Systems Inc.) was used to dry the arrays, and a SureScan high-resolution DNA microarray scanner (Agilent Technologies, CA) was used to scan the arrays. NimbleScan software (Roche NimbleGen, IN) was then used to quantify the images, and JMP Genomics 5.1 software (SAS Institute, Cary, NC, United States) was used for statistical analyses as described previously (Yang et al., 2010). Briefly, raw data were log_2_ transformed before importing into JMP Genomics for analysis followed by a quality control step of data distribution and correlation analyses. The sources of variation including strain, furfural treatment, and technical factors such as array were then visualized by the overlaid kernel density estimates derived from the distribution analysis. The LOWESS algorithm within JMP Genomics was then used for data normalization with the subsequent ANOVA (analysis of variance) analysis conducted to examine differential expression levels between conditions and time points using the false discovery rate (FDR) testing method (*p* < 0.05). Microarray studies have been deposited in NCBI GEO database under the accession number of GSE63540. The interactions among differentially expressed genes were investigated using the String 9.05 pre-computed database (Jensen et al., 2009), available at http://string. embl.de/.

### Next-Generation Sequencing Based Directional mRNA-Seq and Data Analysis

Total RNA used for microarray were also used for directional mRNA-Seq library construction and then sequenced using Illumina HiSeq2000 sequencer. The mRNA-Seq quality was checked using FastQC software (Babraham Bioinformatics, United Kingdom). The reads were then imported into CLC Genomics Workbench 4.1 software (CLC Bio, Denmark) to calculate the RPKM (reads mapping to the genome per kilobase of transcript per million reads sequenced) of each gene. The RPKM value of each gene was then imported into JMP Genomics 5.1 for statistical analysis.

## Data Availability Statement

Microarray studies have been deposited in NCBI GEO database under the accession number of GSE63540. pJL130-Sp has been deposited into GenBank with the accession number of MN723559.

## Author Contributions

MZ led the project. SY, MF, Y-CC, and MZ designed the experiment. MF generated the HPLC data. SY carried out the fermentation, did the RNA extraction, and RNA-Seq data analysis. SB’s lab conducted DNA microarray studies. SY, XM, YH, and MZ analyzed the data. XW and SY wrote the manuscript. PP conducted extensive review. MF, SB, and MZ provided comments for revision. All authors read and approved the final manuscript.

## Conflict of Interest

The authors declare that the research was conducted in the absence of any commercial or financial relationships that could be construed as a potential conflict of interest.

## Footnotes

1 http://www.softberry.com/berry.phtml?topic=bprom&group=programs&subgroup=gfindb
