# Supplementary material for: Transcriptomic Profiles of Zymomonas mobilis 8b to Furfural Acute and Long-Term Stress in Both Glucose and Xylose Conditions
Source: Front Microbiol. 2020 Jan 23;11:13. doi: 10.3389/fmicb.2020.00013 (PMC6989614; doi:10.3389/fmicb.2020.00013)
Supplement: Supplementary file 1 [file Data_Sheet_1.docx]

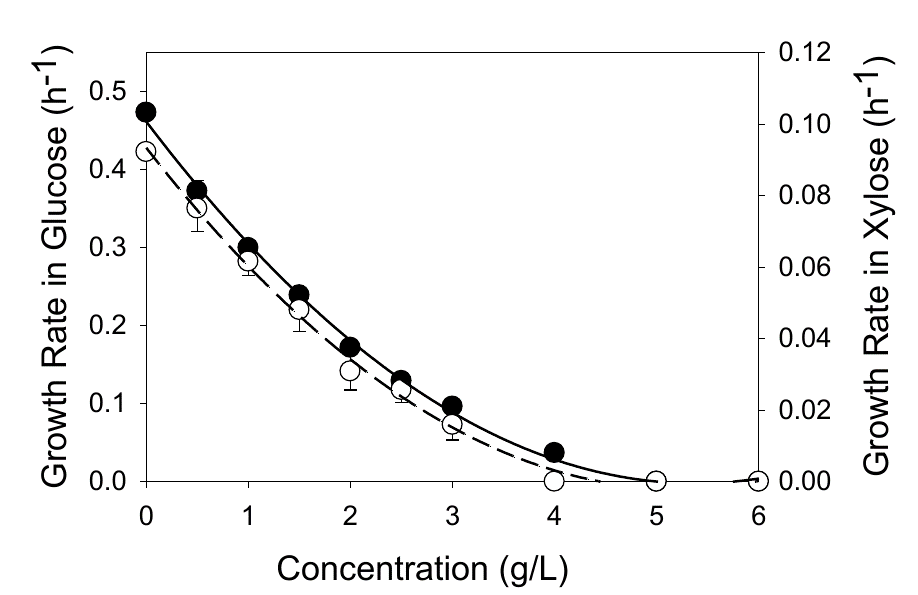


**Fig. S1.** The correlations of furfural concentrations (X-axis, g/L) and growth rates of *Z. mobilis* 8b when glucose (filled black color circle) or xylose (white color circle) was used as carbon source.


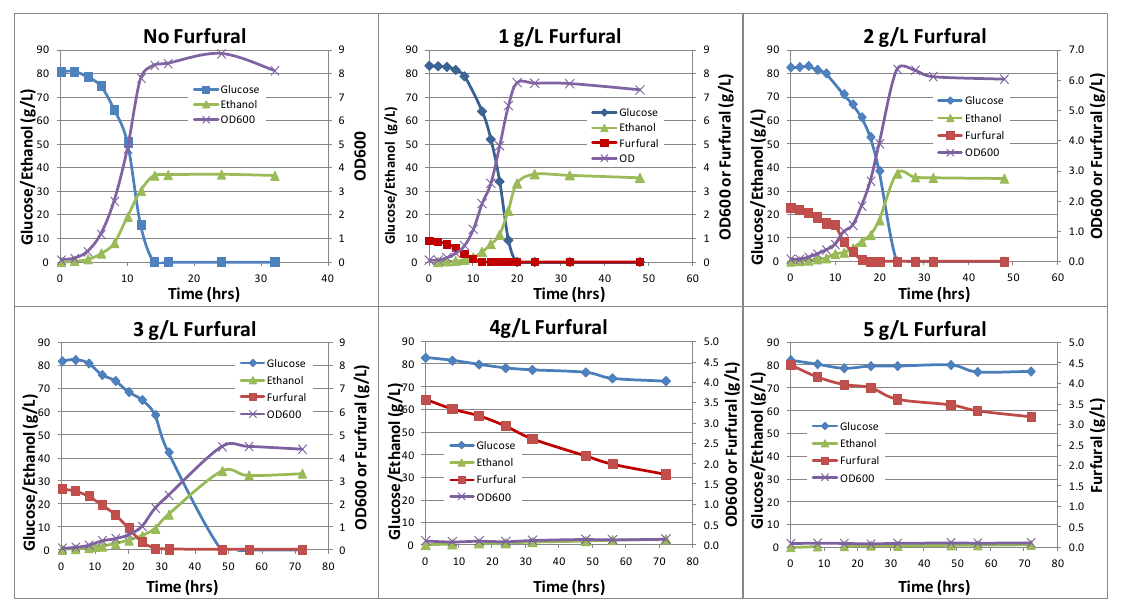


**Fig. S2.** Fermentation performance of *Z. mobilis* 8b with the supplementation of 0 to 5 g/L furfural in pH controlled fermentors (pH6.0, 30^o^C) with 8% (w/v) glucose as the carbon source.

A
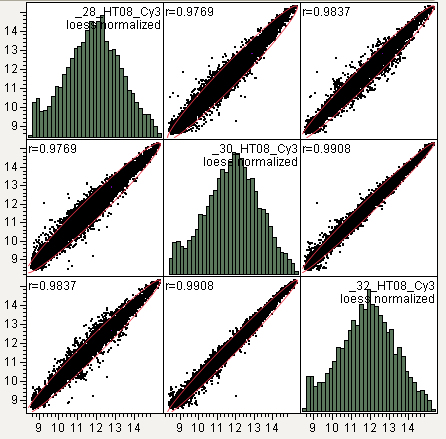
 B
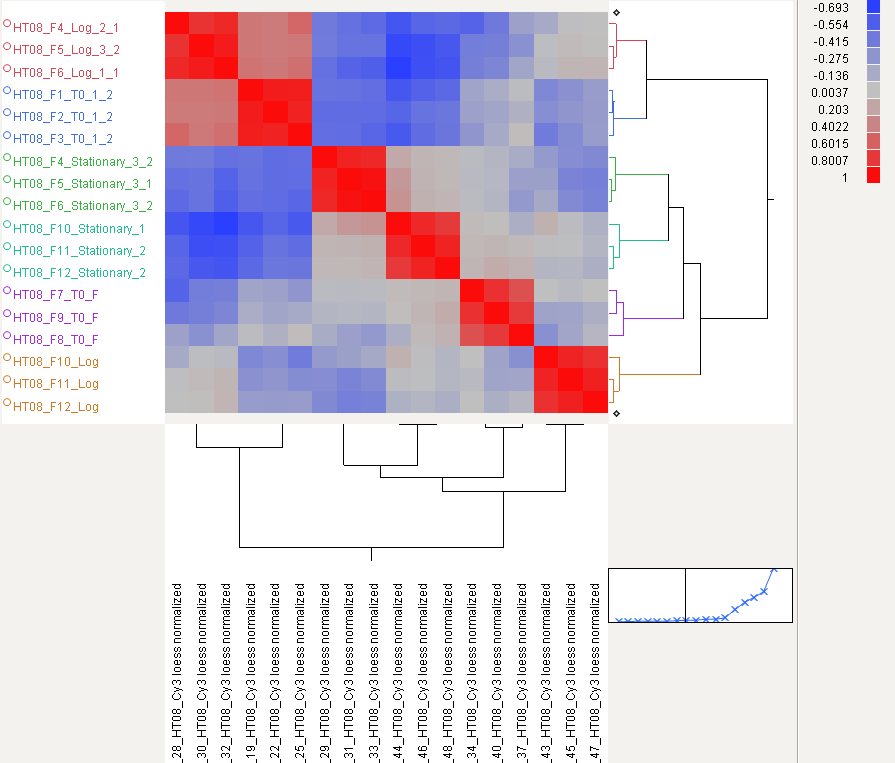


**C**


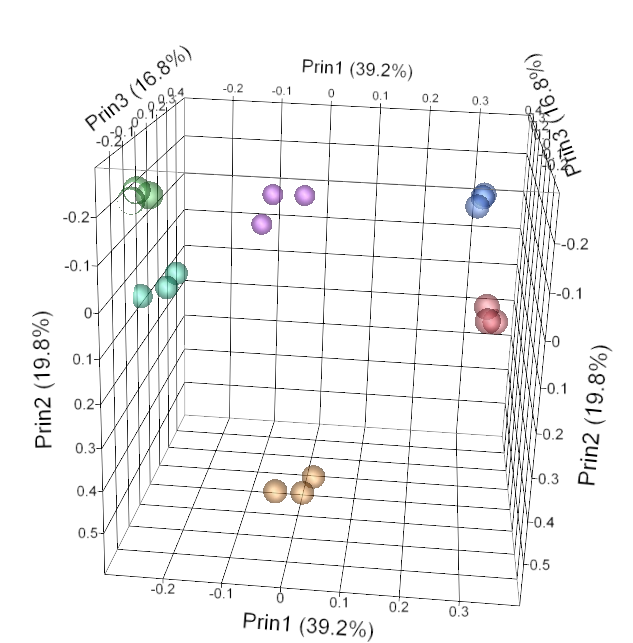


**F10-12: Xyl_Furfural_Log**

**F7-9: Xyl_None_Log**

**F1-3: Gluc_None_Log**

**F4-6: Gluc_Furfural_Log**

**F4-6: Gluc_Furfural_Stat**

**F10-12: Xyl_Furfural_Stat**

**Fig. S3. The quality control analyses of microarray data. A:** Example of correlations among biological replicates of log phase furfural stress samples in RMG8; **B:** Example of hierarchical clustering Heat-Map result among all furfural stress samples; **C:** Example of principal components analysis (PCA) results among all furfural stress samples. Xyl: xylose, Gluc: glucose; None: no furfural supplemented; F1-12: different fermentor number; Log: log phase; Stat: stationary phase.


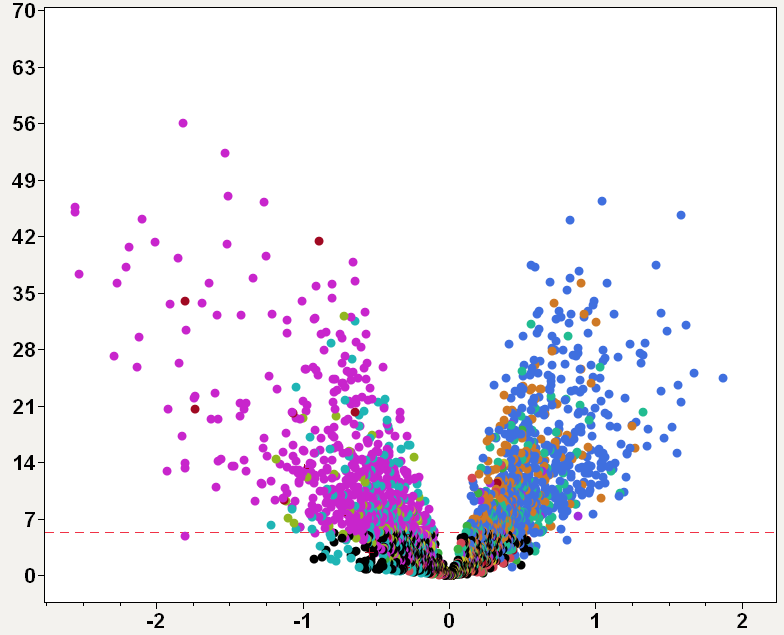

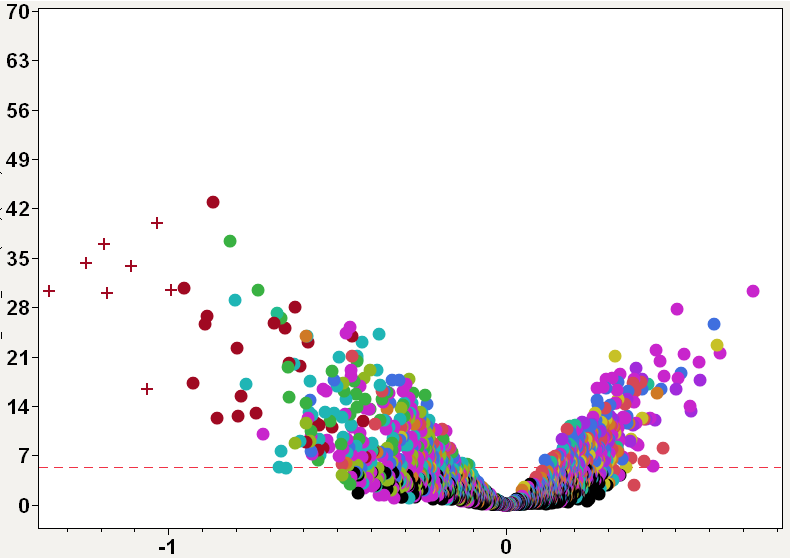

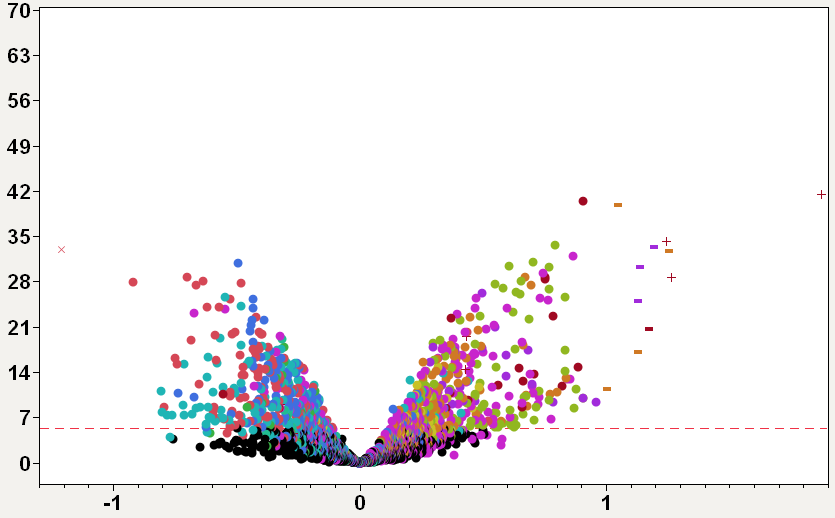

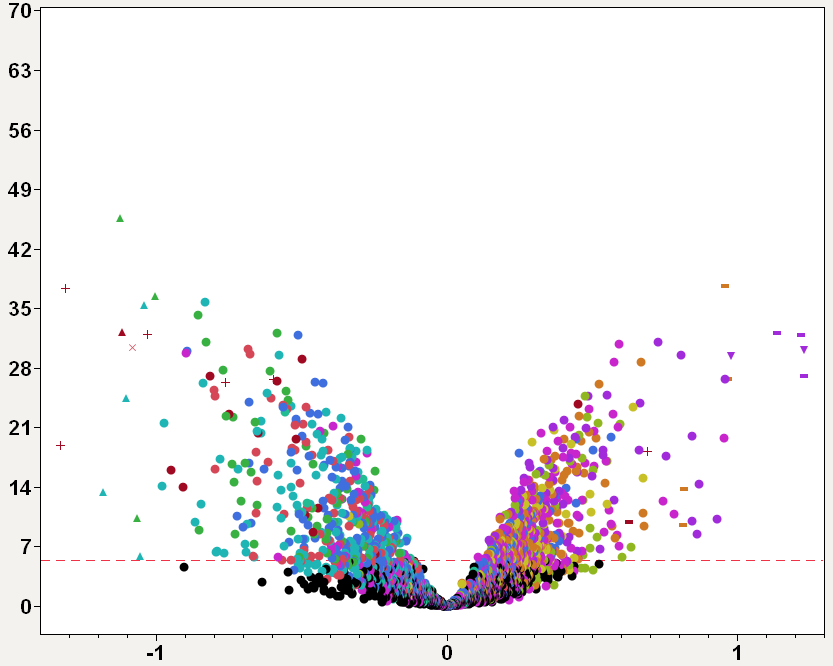


**A: Xylose Vs Glucose**

**B: 0 min Vs 15 min**

**(Furfural Shock)**

**C: 15 min Vs 60 min**

**(Furfural Shock)**

**D: 0 min Vs 60 min**

**(Furfural Shock)**

**Fig. S4. Volcano plots of furfural shock response microarray data using JMP Genomics.** **A:** Sugar effect of glucose versus xylose with and without furfural shock at all the time points of 0, 15 and 60 min; **B-D:** Time course response from 0 min to 15 min, 15 min to 60 min, and 0 min to 60 min with furfural (3 g/L) shock in exponential phase. Each dot represents a genetic feature (either a gene or an intergenic region). Dots above the red dash line are statistically significant features between control and treatment condition. X-axis indicates the log_2_-based ratio between control and treatment condition. Y-axis indicates the statistical significance p-value of –log_10_(P-value).


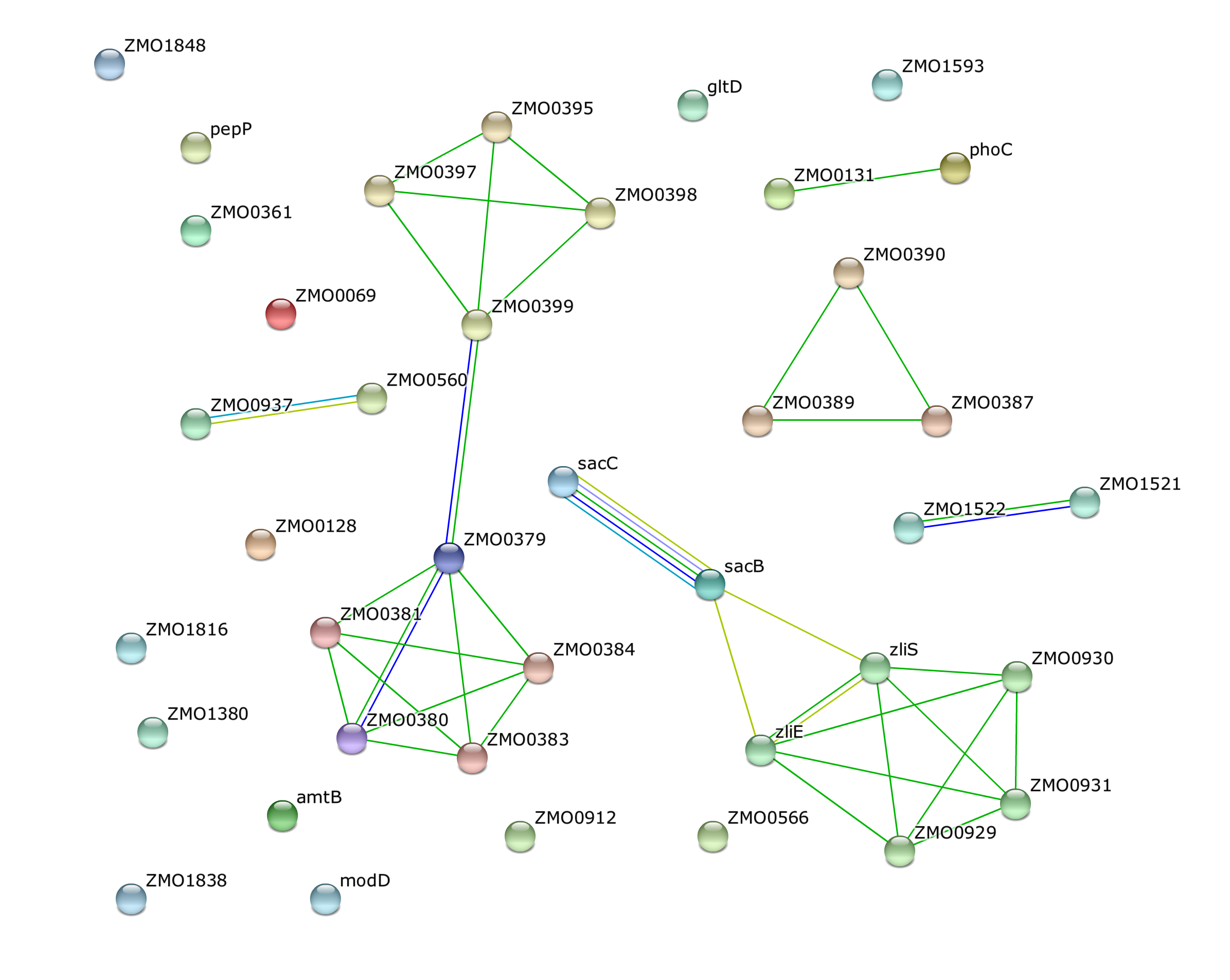


**Fig. S5. Interactions among 39 furfural downregulated genes with at least 2-fold increase in log phase for *Z. mobilis* 8b in both RMG8 and RMX8 in long-term furfural stress experiment.** Data from **Table S4-3, S4-4** were analyzed using Strings pre-computed protein-interaction database. Greater numbers of lines are associated with increased connections and greater confident for associations. The network nodes are proteins. The edges represent the predicted functional associations. An edge may be drawn with up to 7 differently colored lines - these lines represent the existence of the seven types of evidence used in predicting the associations. A red line indicates the presence of fusion evidence; a green line - neighborhood evidence; a blue line - concurrence evidence; a purple line - experimental evidence; a yellow line - textmining evidence; a light blue line - database evidence; a black line - coexpression evidence.


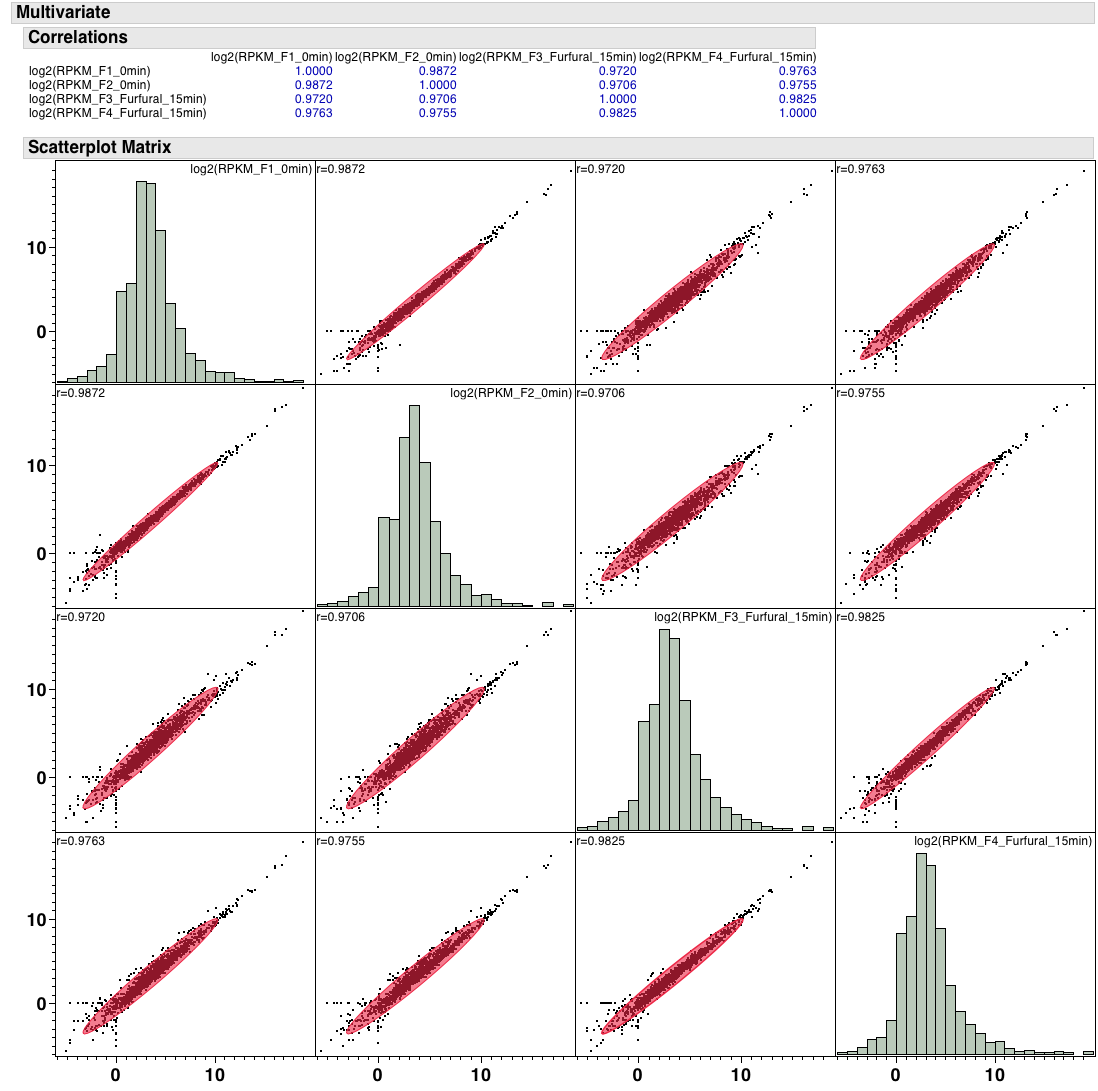


**Fig. S6. Correlations among four datasets generated by directional mRNA-Seq.** The RPKM values based on mapping results to all the genetic features used for microarray study using CLC Genomics Workbench 4.7 were plotted and the correlations among them were shown.


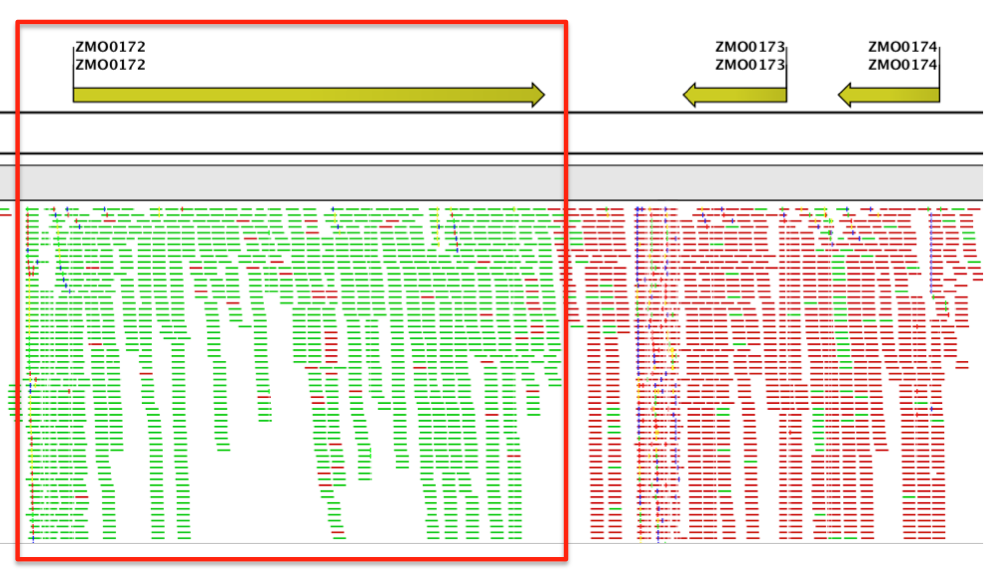

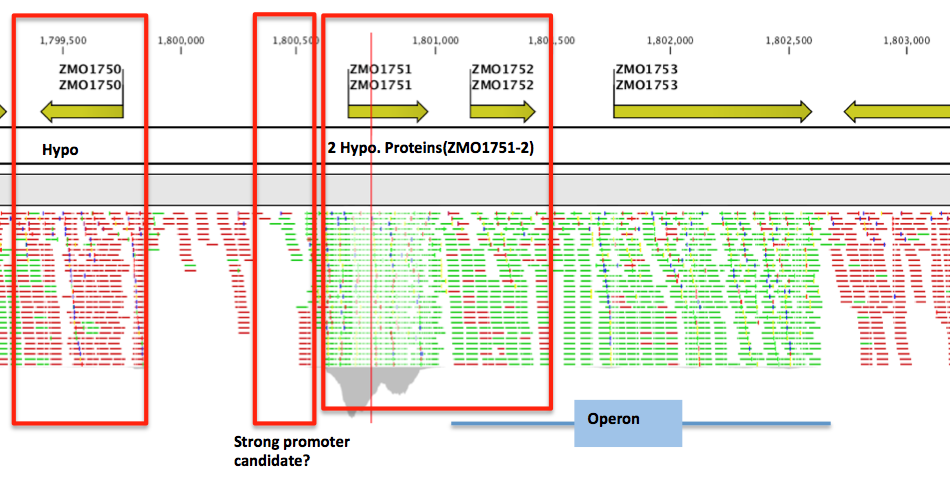


**A**

**B**

**C**

**D**


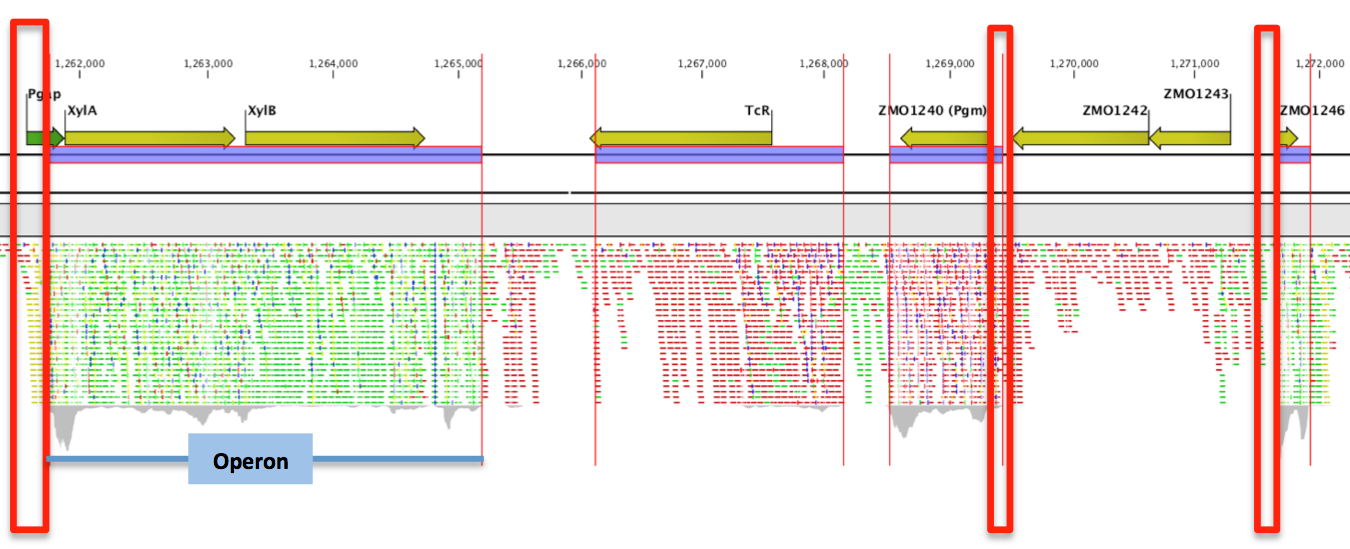

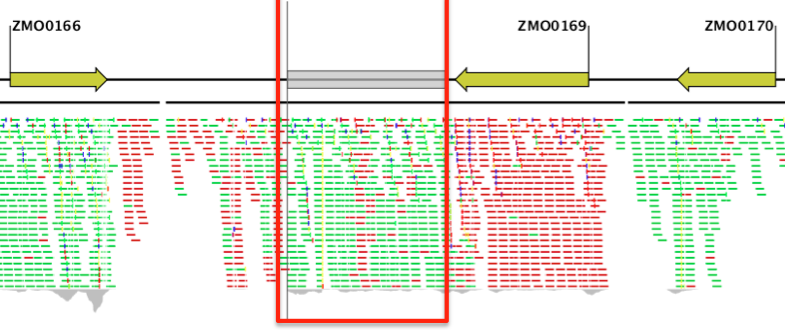


**Fig. S7. The application of directional mRNA-Seq data on transcriptional architecture improvement.** **A:** Transcription start site identification and ORF orientation verification (red box part); **B:** unannotated genetic feature and its orientation identification; **C:** Operon confirmation and Pgap promoter (red box part); **D:** Potential strong promoter and the expression of hypothetic genes (red box) as well as an operon candidate. Each small line is a read mapped to reference sequence, red one indicates the reverse orientation and green one has forward orientation.


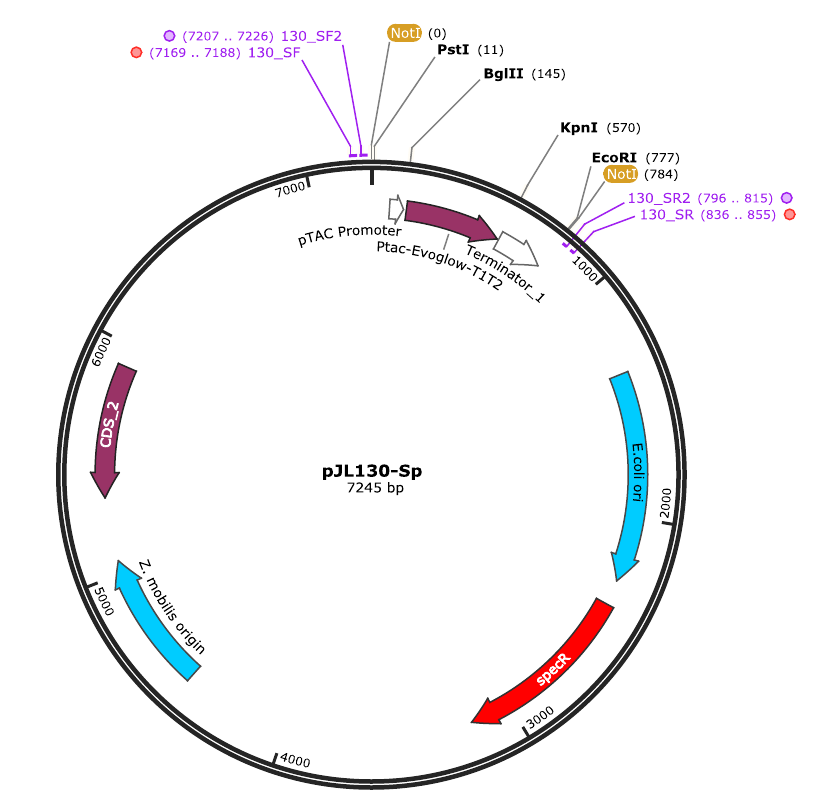


**Fig. S8.** **The plasmid map of pJL130-Sp, which was used as the backbone to replace pTAC**

**promoter and Evoglow coding gene with the target gene and its native promoter.** The fasta

file of plasmid is listed below.

**>pJL130-Sp**

GGCCGCCTGCAGCCGTTCTGGATAATGTTTTTTGCGCCGACATCATAACGGTTCTGGC

AAATATTCTGAAATGAGCTGTTGACAATTAATCATCGGCTCGTATAATGTGTGGAAT

TGTGAGCGGATAACAATTTCACACAGGAGAGATCTTATGGCCTCTTTTCAGTCTTTTG

GTATTCCGGGTCAGTTGGAAGTCATTAAAAAAGCCTTGGATCATGTTCGTGTTGGCG

TTGTTATTACCGATCCTGCCTTGGAAGATAATCCGATTGTTTATGTCAATCAGGGCTT

TGTTCAGATGACCGGTTATGAAACCGAAGAAATCTTGGGTAAAAATGCCCGTTTTTT

GCAGGGTAAACATACCGATCCGGCTGAAGTTGATAATATTCGTACCGCCTTGCAGAA

TAAAGAACCGGTTACCGTTCAGATTCAGAATTATAAAAAAGATGGCACCATGTTTTG

GAATGAACTGAATATTGATCCGATGGAAATTGAAGATAAAACCTATTTTGTCGGCAT

TCAGAATGATATTACCAAACAGAAAGAATATGAAAAATTGCTTGAATAAGGTACCA

GGCATCAAATAAAACGAAAGGCTCAGTCGAAAGACTGGGCCTTTCGTTTTATCTGTTGTTTGTCGGTGAACGCTCTCCTGAGTAGGACAAATCCGCCGGGAGCGGATTTGAACG

TTGCGAAGCAACGGCCCGGAGGGTGGCGGGCAGGACGCCCGCCATAAACTGCCAGG

CATCAAATTAAGCAGAAGGCCATCCTGACGGATGGAATTCGCGGCCGCTAATTCCGG

ATGAGCATTCATCAGGCGGGCAAGAATGTGAATAAAGGCCGGATAAAACTTGTGCT

TATTTTTCTTTACGGTCTTTAAAAAGGCCGTAATATCCAGCTGAACGGTCTGGTTATA

GGTACATTGAGCAACTGACTGAAATGCCTCAAAATGTTCTTTACGATGCCATTGGGA

TATATCAACGGTGGTATATCCAGTGATTTTTTTCTCCATTTTAGCTTCCTTAGCTCCTG

AAAATCTCGATAACTCAAAAAATACGCCCGGTAGTGATCTTATTTCATTATGGTGAA

AGTTGGAACCTCTTACGTGCCGATCAACGTCTCATTTTCGCCAAAAGTTGGCCCAGG

GCTTCCCGGTATCAACAGGGACACCAGGATTTATTTATTCTGCGAAGTGATCTTCCGT

CACAGGTATTTATTCGGCGCAAAGTGCGTCGGGTGATGCTGCCAACTTACTGATTTA

GTGTATGATGGTGTTTTTGAGGTGCTCCAGTGGCTTCTGTTTCTATCAGCTGTCCCTC

CTGTTCAGCTACTGACGGGGTGGTGCGTAACGGCAAAAGCACCGCCGGACATCAGC

GCTAGCGGAGTGTATACTGGCTTACTATGTTGGCACTGATGAGGGTGTCAGTGAAGT

GCTTCATGTGGCAGGAGAAAAAAGGCTGCACCGGTGCGTCAGCAGAATATGTGATA

CAGGATATATTCCGCTTCCTCGCTCACTGACTCGCTACGCTCGGTCGTTCGACTGCGG

CGAGCGGAAATGGCTTACGAACGGGGCGGAGATTTCCTGGAAGATGCCAGGAAGAT

ACTTAACAGGGAAGTGAGAGGGCCGCGGCAAAGCCGTTTTTCCATAGGCTCCGCCCC

CCTGACAAGCATCACGAAATCTGACGCTCAAATCAGTGGTGGCGAAACCCGACAGG

ACTATAAAGATACCAGGCGTTTCCCCCTGGCGGCTCCCTCGTGCGCTCTCCTGTTCCT

GCCTTTCGGTTTACCGGTGTCATTCCGCTGTTATGGCCGCGTTTGTCTCATTCCACGC

CTGACACTCAGTTCCGGGTAGGCAGTTCGCTCCAAGCTGGACTGTATGCACGAACCC

CCCGTTCAGTCCGACCGCTGCGCCTTATCCGGTAACTATCGTCTTGAGTCCAACCCGG

AAAGACATGCAAAAGCACCACTGGCAGCAGCCACTGGTAATTGATTTAGAGGAGTT

AGTCTTGAAGTCATGCGCCGGTTAAGGCTAAACTGAAAGGACAAGTTTTGGTGACTG

CGCTCCTCCAAGCCAGTTACCTCGGTTCAAAGAGTTGGTAGCTCAGAGAACCTTCGA

AAAACCGCCCTGCAAGGCGGTTTTTTCGTTTTCAGAGCAAGAGATTACGCGCAGACC

AAAACGATCTCAAGAAGATCATCTTATTAATCAGATAAAATATTTCTAGATTTCAGT

GCAATTTATCTCTTCAAATGTAGCACCTGAAGTCAGCCCCATACGATATAAGTTGTA

ATTCTCATGTTTGACAGCTTATCATCGATGGAGCACAGGATGACGCCTAACAATTCA

TTCAAGCCGACACCGCTTCGCGGCGCGGCTTAATTCAGGAGTTAAACATCATGAGGG

AAGCGGTGATCGCCGAAGTATCGACTCAACTATCAGAGGTAGTTGGCGTCATCGAGC

GCCATCTCGAACCGACGTTGCTGGCCGTACATTTGTACGGCTCCGCAGTGGATGGCG

GCCTGAAGCCACACAGTGATATTGATTTGCTGGTTACGGTGACTGTAAGGCTTGATG

AAACAACGCGGCGAGCTTTGATCAACGACCTTTTGGAAACTTCGGCTTCCCCTGGAG

AGAGCGAGATTCTCCGCGCTGTAGAAGTCACCATTGTTGTGCACGACGACATCATTC

CGTGGCGTTATCCAGCTAAGCGCGAACTGCAATTTGGAGAATGGCAGCGCAATGAC

ATTCTTGCAGGTATCTTCGAGCCAGCCACGATCGACATTGATCTGGCTATCTTGCTGA

CAAAAGCAAGAGAACATAGCGTTGCCTTGGTAGGTCCAGCGGCGGAGGAACTCTTT

GATCCGGTTCCTGAACAGGATCTATTTGAGGCGCTAAATGAAACCTTAACGCTATGG

AACTCGCCGCCCGACTGGGCTGGCGATGAGCGAAATGTAGTGCTTACGTTGTCCCGC

ATTTGGTACAGCGCAGTAACCGGCAAAATCGCGCCGAAGGATGTCGCTGCCGACTG

GGCAATGGAGCGCCTGCCGGCCCAGTATCAGCCCGTCATACTTGAAGCTAGGCAGGC

TTATCTTGGACAAGAAGATCGCTTGGCCTCGCGCGCAGATCAGTTGGAAGAATTTGT

TCACTACGTGAAAGGCGAGATCACCAAGGTAGTCGGCAAATAATGTCTAACAATTCG

TTCAAGCCGACGCCGCTTCGCGGCGCGGCTTAACTCAAGCGTTAGAGAGCTGGGGAA

GACTATGCGCGATCTGTTGAAGGTGGTTCTAAGCCTCGTACTTGCGATGGCATCGGGGCAGGCACTTGCTGACCTGCCAACGCGCCTTTGTAGTCTTGGCCTGTTGTGTGCATGA

GCAAATCAATGGCACCACCCCCTCCTTTTTGAGCTGAATGGTCATAAAATTTATAATT

ATCTATCGTAATTCGGAATCTATGTTCAGGGTCTCGCCATTGCTTTTTGTCTGCTGGG

TCAAGTTCCATGCCTAAGGTTTTTAAGACATCAGAAAGAGGTATTGCACGCATGCTA

TCAGCTTTTCTTCTAGCTAATGACAGGGCTTCCTCTGCTCTATCTGCTCGTTTTTTTTC

TTCCACATATCTCGCCGCTTTGTCAGCCAGCGGCTGTATTACGGAAAGTGCCGATTTT

TGGGCTTTTAGGCGTTCTTTTTCTGCCCATTCTTCCTTATTTGTAAAAATTGAGGGTGG

GATGGGTGCCTGAATCTTGGGATCTAGCTGTAAAGTTTTGTTGATATTTCCGTAATGT

CTTTGGACTCTTTGATGCGTTGCTTTTGAACCTTTTACGCCTCTGGCCAGCCCTAGAG

GCTCCATAGAAGCCGCATAATCCGTCTGGAGGGCAGAAAGGGCTTTTCGACCATCAA

ACCATCTCGATGCGTTTAAACGGCCTGTATCGGGGTCTCTAGGCACCATAAAGCCGG

TTAAGTGGGGTGTTGTTTCATCAGCATGTAGCTGAAGAGATACAAGGTTGTTTTCTCC

AAAGGTTTGTTCCGCCCATTGCTGGGTGATTGTTTTCCAGTGTTCGAGTTTTTCAGGA

GTGGCCTGTTTTGACCATTCTGGAGACATACCAAAGAACAGTTCTATGGCCTGCACA

CCGTTTTTTCTAAGAGGCTTTCCCGTTTCTTTCTGAATTTTATTCAGCATAGATTTAAC

ATCTGCTGATGGGTCAGTAGAGCCTTTGAGTATTTCGTTTAGTTCTTTTCTATCTGGG

TCAGCGTTTTGTGTTTCGCGGCCTCGCGTCATATGCAGGCTCGCGGCTTTAATCGTGC

CAACTGTTTTATGTTTTTCAAACCTAAAGATTGCATAGTTCGGCATGTTTTAACTGCT

TTAATTTGAGAAAAGACCAGAGGAAATAATCCAGCCTATATTTCTTTCCCTAGTAGC

GAACTGGAATTGTTTTTCCGAAGGAAAAAAGCAATTCCGTAGTGAGTACTGAATTTA

TTCTGATTCGTCTTGCTTTTGGAGCGTCTTTTTGCGTTCTATAACTGTTGTGAAAGCTA

CGCGGTCGCCATTGAAAACGAAATTAGGATTAATAAAATACCATCCTTGGCGAACAT

GCTTTGCAATGATTTTAGCTTTTTCTAATTCGGCTAGACCTCTTGCAAAGGTAGCTTG

AGATAGTGCCAGTTTTTTTTCTTGTGCGTTAAGAAAGTCCTCTAAAACGAATTTGTCT

AAAGGGACGAGGTCTTTGCTGATGCCTTTGTCTTGAAGTATCCAAACCAGAACGCTG

AAAGCTTTTATTCCAGCGGCTCCTAGTTCAAAAGTTAGCGCGATATTGGTGCTAAAT

AATTTTACAAATTCTTCACTATCAACACGTCTGTAAGTCGTCACATGAGTGCCTTGCA

TCTCACCAGTGGCTTGATTGACCAGAATGTTATCATCTCGTCCTAATCGAGATAACTG

AACCCTCTGACTTTTAACTGGCACAACCATACCTTCGATGAAAGGATTCTCGTCATAT

CTGATTGGCTGCTTTCTCAATTTTGTCGCCATATTTGATAAACCTTTAATCAAAAAAA

CCACATTTTTTGATTATACCTATTCATCGAATGAGGCAAGGTCTATCAATTTTACCCC

TTTTTTTGATAGACGGTTTAATCAATATTGATAGACCCCTTCACAGATTCTGAAAATC

GACTTCCCTATTTTAGGGATATTTTCACGATTCCCTTTCTTAGTTCTTCCTAGTGGGGA

AATTCGTTGAATCCTGCCTCGGAAAAACCATGAGAAAGCTGTTGGTTATATACACGG

GCAAAGCCACCCTATTTTTAGCTACTGGGGAAAGAGATAAGGCAGGGTATTTGTAAA

ATTAAAACCGGATTTTTCGCTTTACGGTTTGTTTAGGCGCAACTGTCTTTTTAAGACC

GCGTTTAACCATCAAAAGATCGTTCCAATCTTTTCCGTGTATCATCTGTTCTTTAGGT

GGGAGCCAGTTTTCAACTTTTTTTGTTGGAAACGCGGCTTTAATCGCTCCGACTAATA

GCGATGCTGCTCTTTGTCCTACAGCATCCCAATCATAGGCAATATGGACAGAAGATG

CCTTTTCAACGATTTTTCGGAGAGTTTTAGTAAGAGACGTTCTTACGCCGCTGGTGCT

TAATAATTTTACGCCAGCTTTAATTTTTTCTGGGCTTAAAAAGCCGACTACTGAAATC

GCGTCTATCGCACTTTCAGCGATATAAAGATCATACTTTTCGTCATTTTTTACATTGA

TGCTGCCAGTAAAATGGGCTTCGCGACTGCTTCCCAAGGCTAACCCTTTAAAACCAC

TGCTTGTTCCGCGTAATTCTGCGCCCTGAAGTGTATCTTTATCGTCATACATCAAGAA

GGCTACATTACCGCGATCATCTGTTCGGATAGAGTCAGGAATATTGTTAAATGATAT

TCCTCGGGCAGCGTTGGGTCCTGGCCACGGGTGCGCATGATCGTGCTCCTGTCGTTG

AGGACCCGGCTAGGCTGGCGGGGTTGCCTTACTGGTTAGCAGAATGAATCACCGATACGCGAGCGAACGTGAAGCGACTGCTGCTGCAAAACGTCTGCGACCTGAGCAACAAC

ATGAATGGTCTTCGGTTTCCGTGTTTCGTAAAGTCTGGAAACGCGGAAGTCCCCTAC

GTGCTGCTGAAGTTGCCCGCAACAGAGAGTGGAACCAACCGGTGATACCACGATAC

TATGACTGAGAGTCAACGCCATGAGCGGCCTCATTTCTTATTCTGAGTTACAACAGT

CCGCACCGCTGTCCGGTAGCTCCTTCCGGTGGGCGCGGGGCATGACTATCGTCGCCG

CACTTATGACTGTCTTCTTTATCATGCAACTCGTAGGACAGGTGCCGGCAGCGCCCA

ACAGTCCCCCGGCCACGGGGCCTGCCACCATACCCACGCCGAAACAAGCGCCCTGC

ACCATTATGTTCCGGATCTGCATCGCAGGATGCTGCTGGCTACCCTGTGGAACACCT

ACATCTGTATTAACGAAGCGCTAACCGTTTTTATCAGGCTCTGGGAGGCAGAATAAA

TGATCATATCGTCAATTATTACCTCCACGGGGAGAGCCTGAGCAAACTGGCCTCAGG

CATTTGAGAAGCACACGGTCACACTGCTTCCGGTAGTCAATAAACCGGTAAACCAGC

AATAGACATAAGCGGCTATTTAACGACCCTGCCCTGAACCGACGACCGGGTCGAATT

TGCTTTCGAATTTCTGCCATTCATCCGCTTATTATCACTTATTCAGGCGTAGCACCAG

GCGTTTAAGGGCACCAATAACTGCCTTAAAAAAATTACGCCCCGCCCTGCCACTCAT

CGCAGTACTGTTGTAATTCATTAAGCATTCTGCCGACATGGAAGCCATCACAGACGG

CATGATGAACCTGAATCGCCAGCGGCATCAGCACCTTGTCGCCTTGCGTATAATATT

TGCCCATGGTGAAAACGGGGGCGAAGAAGTTGTCCATATTGGCCACGTTTAAATCAA

AACTGGTGAAACTCACCCAGGGATTGGCTGAGACGAAAAACATATTCTCAATAAAC

CCTTTAGGGAAATAGGCCAGGTTTTCACCGTAACACGCCACATCTTGCGAATATATG

TGTAGAAACTGCCGGAAATCGTCGTGGTATTCACTCCAGAGCGATGAAAACGTTTCA

GTTTGCTCATGGAAAACGGTGTAACAAGGGTGAACACTATCCCATATCACCAGCTCA

CCGTCTTTCATTGCCATACGGAATTAGC
