# Supplementary material for: Transcriptomic Profiles of Zymomonas mobilis 8b to Furfural Acute and Long-Term Stress in Both Glucose and Xylose Conditions
Source: Front Microbiol. 2020 Jan 23;11:13. doi: 10.3389/fmicb.2020.00013 (PMC6989614; doi:10.3389/fmicb.2020.00013)
Supplement: Supplementary file 2 [file Table_1.docx]

**Table S1. The information of array data used for furfural stress response and time course shock response transcriptomic profiling. Exp.:** stress or time course experiment and the arrays associated with each analysis; **F#:** fermentor number.

| **Exp.** | **Sample Description** | **Sugar** | **Treatment** | **Time Phase** | **F#** |
| --- | --- | --- | --- | --- | --- |
| Time course | HT08_F1_T15min_1_2 | Glucose | Furfural | 15 min_log | F1 |
|  | HT08_F2_T15min_1_2 | Glucose | Furfural | 15 min_log | F2 |
|  | HT08_F3_T15min_1_2 | Glucose | Furfural | 15 min_log | F3 |
|  | HT08_F1_T60min_1_2 | Glucose | Furfural | 60 min_log | F1 |
|  | HT08_F2_T60min_1_2 | Glucose | Furfural | 60 min_log | F2 |
|  | HT08_F3_T60min_1_2 | Glucose | Furfural | 60 min_log | F3 |
|  | HT08_F7_T15min_F | Xylose | Furfural | 15 min_log | F7 |
|  | HT08_F8_T15min_F | Xylose | Furfural | 15 min_log | F8 |
|  | HT08_F9_T15min_F | Xylose | Furfural | 15 min_log | F9 |
|  | HT08_F7_T60min_F | Xylose | Furfural | 60 min_log | F7 |
|  | HT08_F8_T60min_F | Xylose | Furfural | 60 min_log | F8 |
|  | HT08_F9_T60min_F | Xylose | Furfural | 60 min_log | F9 |
| Stress and Time course | HT08_F1_T0_1_2 | Glucose | None | 0 min_log | F1 |
|  | HT08_F2_T0_1_2 | Glucose | None | 0 min_log | F2 |
|  | HT08_F3_T0_1_2 | Glucose | None | 0 min_log | F3 |
|  | HT08_F7_T0_F | Xylose | None | 0 min_log | F7 |
|  | HT08_F8_T0_F | Xylose | None | 0 min_log | F8 |
|  | HT08_F9_T0_F | Xylose | None | 0 min_log | F9 |
| Stress | HT08_F4_Log_2_1 | Glucose | Furfural | log | F4 |
|  | HT08_F5_Log_3_2 | Glucose | Furfural | log | F5 |
|  | HT08_F6_Log_1_1 | Glucose | Furfural | log | F6 |
|  | HT08_F4_Stationary_3_2 | Glucose | Furfural | Stationary | F4 |
|  | HT08_F5_Stationary_3_1 | Glucose | Furfural | Stationary | F5 |
|  | HT08_F6_Stationary_3_2 | Glucose | Furfural | Stationary | F6 |
|  | HT08_F10_Log | Xylose | Furfural | log | F10 |
|  | HT08_F11_Log | Xylose | Furfural | log | F11 |
|  | HT08_F12_Log | Xylose | Furfural | log | F12 |
|  | HT08_F10_Stationary_1 | Xylose | Furfural | Stationary | F10 |
|  | HT08_F11_Stationary_2 | Xylose | Furfural | Stationary | F11 |
|  | HT08_F12_Stationary_2 | Xylose | Furfural | Stationary | F12 |
